# Coordinated infraslow neural and cardiac oscillations mark fragility and offline periods in mammalian sleep

**DOI:** 10.1126/sciadv.1602026

**Published:** 2017-02-08

**Authors:** Sandro Lecci, Laura M. J. Fernandez, Frederik D. Weber, Romain Cardis, Jean-Yves Chatton, Jan Born, Anita Lüthi

**Affiliations:** 1 Department of Fundamental Neurosciences, University of Lausanne, 1005 Lausanne, Switzerland.; 2 Institute of Medical Psychology and Behavioral Neurobiology, University of Tübingen, 72076 Tübingen, Germany.

**Keywords:** mammalian sleep, sleep quality, sleep architecture, arousability, memory consolidation, sleep spindles, hippocampal ripples, declarative memory, sensory processing, autonomic system

## Abstract

Rodents sleep in bouts lasting minutes; humans sleep for hours. What are the universal needs served by sleep given such variability? In sleeping mice and humans, through monitoring neural and cardiac activity (combined with assessment of arousability and overnight memory consolidation, respectively), we find a previously unrecognized hallmark of sleep that balances two fundamental yet opposing needs: to maintain sensory reactivity to the environment while promoting recovery and memory consolidation. Coordinated 0.02-Hz oscillations of the sleep spindle band, hippocampal ripple activity, and heart rate sequentially divide non–rapid eye movement (non-REM) sleep into offline phases and phases of high susceptibility to external stimulation. A noise stimulus chosen such that sleeping mice woke up or slept through at comparable rates revealed that offline periods correspond to raising, whereas fragility periods correspond to declining portions of the 0.02-Hz oscillation in spindle activity. Oscillations were present throughout non-REM sleep in mice, yet confined to light non-REM sleep (stage 2) in humans. In both species, the 0.02-Hz oscillation predominated over posterior cortex. The strength of the 0.02-Hz oscillation predicted superior memory recall after sleep in a declarative memory task in humans. These oscillations point to a conserved function of mammalian non-REM sleep that cycles between environmental alertness and internal memory processing in 20- to 25-s intervals. Perturbed 0.02-Hz oscillations may cause memory impairment and ill-timed arousals in sleep disorders.

## Teaser

Mammalian sleep alternates between sensory-responsive and protected offline periods via coordinated neural and cardiac rhythms.

## INTRODUCTION

All mammals benefit from sleep in fundamental aspects for brain and body (*1*, *2*). For sleep to be beneficial, it must be of sufficient duration and physiological continuity. Conversely, sleep needs to retain a certain degree of fragility, because all sleeping organisms remain capable of a behavioral arousal response to salient stimuli and potential threats. To date, it is unclear how sleep generates advantageous effects while maintaining sensory responsiveness and how the two opposite needs for continuity and fragility are balanced. Recently, given the enormous differences in sleep fragmentation between mammalian species (*3*), the idea of universal beneficial functions of sleep for all mammals has even been challenged (*4*).

Ongoing electrical rhythms in the thalamocortical loops of the sleeping brain are central to disrupt sensory information processing. Among these, sleep spindles are particularly efficient in attenuating the likelihood that sensory stimuli arrive in cortex (*5*, *6*). Spindles are electroencephalographic hallmarks of non–rapid eye movement (non-REM) sleep in the sigma (10 to 15 Hz) power range that occur preferentially during human “light” sleep (*7*) and that last for ~0.5 to 3 s throughout mammals (*8*). Sensory processing thus varies momentarily along with the spectral dynamics of thalamocortical rhythms and contributes to sleep fragility (*9*). Non-REM sleep is also accompanied by marked changes in the autonomic system, notably including decreases in heart rate that recover before transitions to REM sleep or awakening (*10*). Therefore, periods of sleep fragility, during which awakenings are more likely to occur, should involve the autonomic system. To date, however, an analysis of sleep fragility periods based on a combined assessment of sensory processing, spectral dynamics, and autonomic parameters has not been carried out. Moreover, how fragility phases interchange with phases of continuity and how these concur with hallmarks of memory processing during sleep remain open questions.

## RESULTS

### Undisturbed non-REM sleep in mice shows a 0.02-Hz oscillation in sigma power

**Fig. 1:**
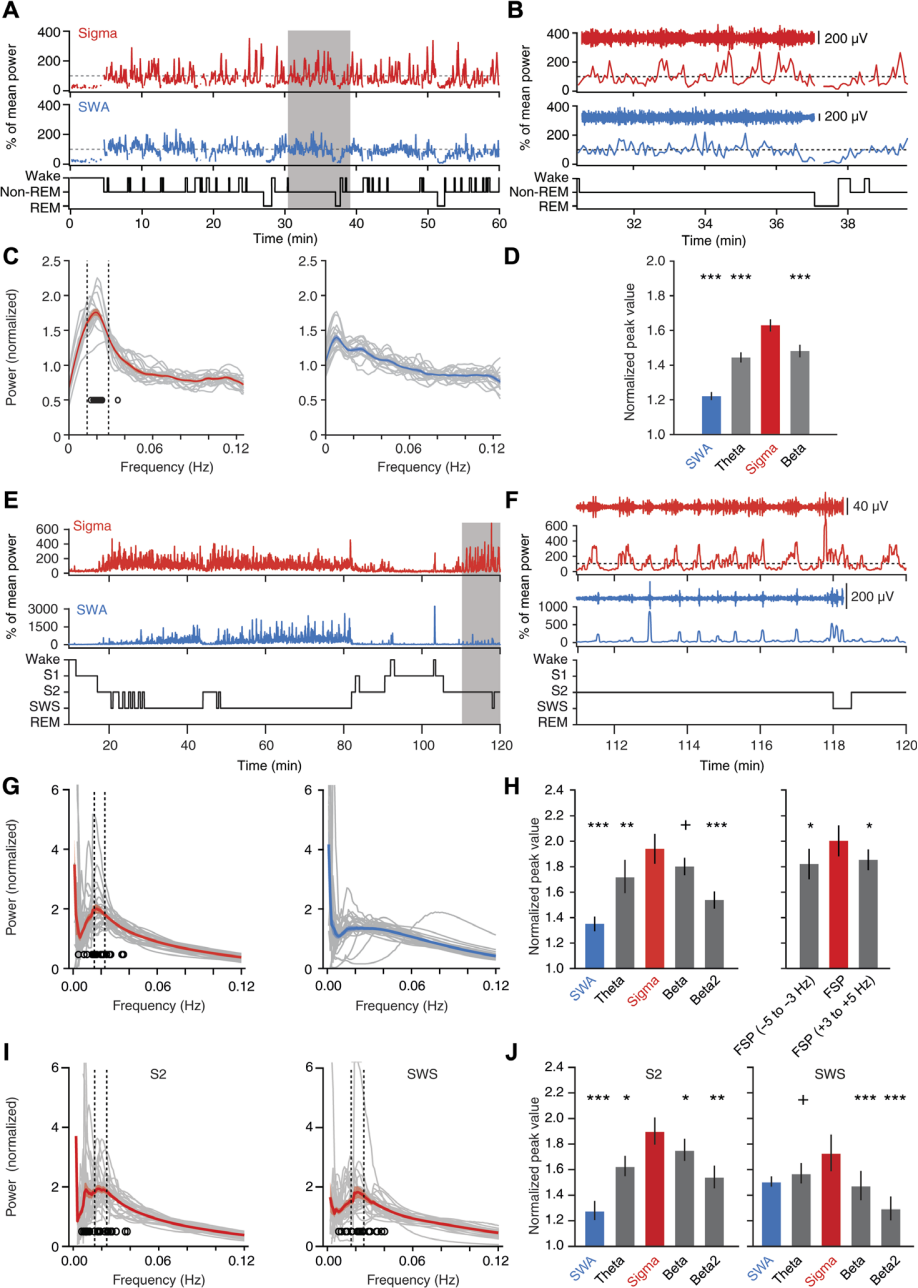
The 0.02-Hz oscillation in sigma power in undisturbed non-REM sleep of mice and humans. (**A** to **D**) Sleep analysis in freely moving mice (*n =* 18). (A and B) Sigma (red; 10 to 15 Hz) and SWA (blue; 0.75 to 4 Hz) power time course for a single mouse, with hypnograms shown below. Gray-shaded area in (A) is expanded in (B), with aligned band-pass–filtered ECoG traces. (C) FFT of power time course for sigma (left) and SWA (right) for individual mice (gray traces, *n =* 18) and for the average across mice (color + shading, means ± SEM). Open circles denote FFT peaks obtained from Gaussian fits (their SD was 0.015 Hz). Vertical dotted lines indicate mean peak frequency ± 0.5 SD. Minor ticks are added to indicate the 0.02-Hz value on the frequency axis. (D) Mean peak values from (C) for sigma power, SWA, theta (6 to 10 Hz), and beta (16 to 20 Hz) bands (Friedman rank sum test; *P* = 4.9 × 10^−8^, post hoc Wilcoxon signed-rank tests relative to sigma power, *P* = 7.63 × 10^−6^ for SWA; *P* = 3.81 × 10^−5^ for theta; *P* = 1.53 × 10^−5^). (**E** to **J**) Sleep analysis in humans (*n =* 27). (E and F) Same as (A) and (B) for a single human subject (sigma, 10 to 15 Hz; SWA, 0.5 to 4 Hz). (G) Power spectral profiles for sigma power and SWA time course during non-REM sleep (S2 + SWS) graphed as in (C). The open circles indicate the peak of Gaussian fits, which show an SD of 0.008 Hz. (H) Left: Same as (D), calculated over all non-REM sleep (theta, 4 to 8 Hz; beta, 16 to 20 Hz; beta2, 20 to 24 Hz). Same statistics as (D): *P* = 3.5 × 10^−4^; post hoc Wilcoxon signed-rank tests with *P* = 9.5 × 10^−6^ for SWA; *P* = 0.009 for theta; *P* = 0.095 for beta; *P* = 4.3 × 10^−4^ for beta2. Right: Individual fast spindle power peaks (FSP ± 1 Hz) and adjacent frequency bands. Same statistics as in (C): *P* = 0.034 for Friedman rank sum test; *P* = 0.015 for −5 to −3 Hz; *P* = 0.032 for +3 to +5 Hz. (I) Same analysis as (G), restricted to S2 or to SWS and sigma power. (J) Left: Power analysis as in (H) restricted to S2 sleep revealed a prominent peak for sigma power over other frequency bands (*n =* 27; Friedman rank sum test, *P* = 8.5 × 10^−6^, followed by Wilcoxon signed-rank tests with respect to sigma power; *P* = 2.5 × 10^−6^ for SWA; *P* = 0.023 for theta; *P* = 0.011 for beta; *P* = 0.002 for beta2). Right: As left for SWS only (same statistics as the left panel: *P* = 1.8 × 10^−4^; *P* = 0.11 for SWA; *P* = 0.052 for theta; *P* = 6 × 10^−4^ for beta; *P* = 1.6 × 10^−4^ for beta2). ^+^*P* < 0.1, *P < 0.05, **P < 0.01, ***P < 0.001.

To examine whether mouse non-REM sleep shows microarchitectural dynamics indicative of variable fragility, we used polysomnography [electroencephalography (EEG)/electrocorticography (ECoG) and electromyography (EMG)] in freely moving mice (*11*) and inspected the temporal evolution of two major spectral bands characteristic for non-REM sleep: the slow-wave activity (SWA; 0.75 to 4 Hz) and the sigma (10 to 15 Hz) power band (*8*). Epochs of non-REM sleep were selected during the first 100 min after onset of the light phase [zeitgeber time 0 (ZT0)], during which mice slept ~63% of their time (*n =* 18 mice). During this period, non-REM sleep occurred in bouts ranging from 8 to >512 s in duration, with a mean length of 108.6 ± 8.1 s. Both sigma power and SWA were elevated during non-REM sleep and decreased during waking or REM sleep (Fig. 1A and fig. S1). Unexpectedly, we noticed that sigma power, but not SWA, displayed marked variations that recurred periodically in both short and long non-REM sleep bouts (Fig. 1B and fig. S1). We assessed the dynamics of sigma power across time for consolidated non-REM sleep periods ≥96 s (mean duration, 180.4 ± 8.8 s) (fig. S2). This revealed a predominant frequency of 0.021 ± 0.001 Hz (Fig. 1C) in a fast Fourier transform (FFT), corresponding to a cycle length of 47.6 ± 2.1 s. In contrast, such a prominent peak was not present for the SWA time course (Fig. 1C), and it was markedly weaker in frequency bands adjacent to the sigma band (*n =* 18; Friedman’s test, *P* = 7.9 × 10^−5^; Fig. 1D). Further analyses and computational simulations confirmed that sigma power oscillated robustly in the 0.02-Hz frequency range (fig. S3). First, a 0.02-Hz oscillation emerged when the analysis was restricted to long non-REM sleep bouts (≥192 s, corresponding to 32.08% of all the bouts ≥96 s) (fig. S3, A to F), demonstrating that sigma power cycles on a 50-s time scale during consolidated non-REM sleep. Second, computational simulations indicated that a true sinusoidal component at ~0.02 Hz rather than scale-free power dynamics underlay the peak in the FFT (fig. S3, G to J). Third, autocorrelations displayed side peaks with a periodicity of 52.6 ± 0.83 s (paired *t* test compared to shuffled data, *t* = 3.82, *P* = 0.0015; fig. S4, A and B). These combined results demonstrate that mouse non-REM sleep contains a 0.02-Hz oscillation of sigma power dynamics, corresponding to a periodicity of ~50 s.

### Undisturbed non-REM sleep in humans shows a 0.02-Hz oscillation in sigma power

To explore whether this infraslow 0.02-Hz oscillation exists in higher mammals, we carried out a comparable power analysis for human sleep (fig. S5). As expected, sigma power was high during stage 2 (S2) sleep (light sleep) and declined during slow-wave sleep (SWS; “deep” sleep) (*7*) when SWA emerged (Fig. 1E and fig. S6). The 0.02-Hz oscillation was present in the sigma power band (10 to 15 Hz) with maximal amplitudes comparable to those in mice (Fig. 1F and fig. S7) but was attenuated in the SWA band (Fig. 1, G and H). When analyzed across all non-REM sleep (S2 + SWS), human sigma power oscillations had a periodicity of 0.019 ± 0.001 Hz (*n =* 27), corresponding to a cycle length of 52.6 ± 2.6 s, comparable to mice. The sigma power band showed the most pronounced dynamics of around 0.02 Hz, while adjacent frequency bands displayed distinctly weaker periodicity (*n* = 27; Friedman’s test, *P* < 3.5 × 10^−4^; Fig. 1H). Furthermore, SWA lacked prominent infraslow dynamics and showed a minor spectral peak (Fig. 1H). In humans, there are fast (12 to 15 Hz) and slow spindles (9 to 12 Hz), with the former being prevalent during S2 and providing a distinct peak in individual power spectra (*12*). When focusing our analysis on the fast spindles, we found that 0.02-Hz oscillations emerged strongest in a 2-Hz band around the fast spindle peak (FSP; 13.16 ± 0.12 Hz) and fell off in adjacent bands (Fig. 1H). The 0.02-Hz oscillation of sigma power appeared to be more prominent in S2 than in SWS (Fig. 1, I and J). Autocorrelations confirmed the oscillatory nature of sigma power dynamics, displaying side peaks with a periodicity of 53.0 ± 2.73 s (Wilcoxon signed-rank test for periodicity, *P* = 0.026; fig. S4, C and D). Together, these data unravel a 0.02-Hz oscillation common to both human and mouse non-REM sleep that is prevalent for sigma power and most prominent for fast spindles in human non-REM sleep.

### The 0.02-Hz oscillation shows regional specificity in both mice and humans

**Fig. 2:**
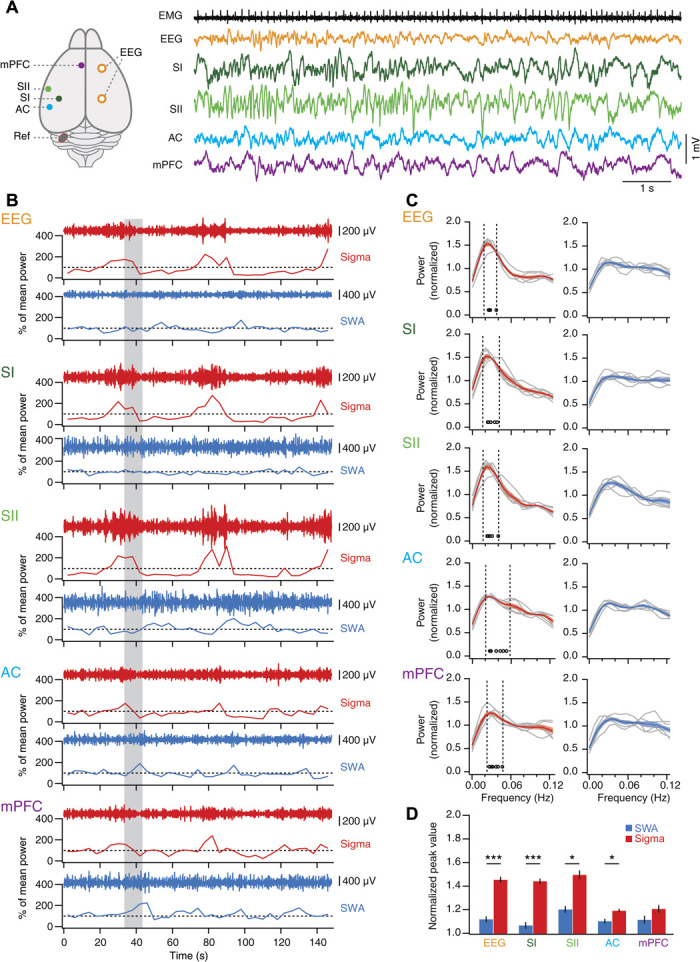
The 0.02-Hz oscillation is present in local cortical areas and predominates in somatosensory cortex. (**A**) Top view of mouse brain with indication of recording sites and with corresponding representative traces obtained during non-REM sleep scored on the basis of EEG/EMG recordings. (**B**) Sigma (red) and SWA (blue) power time course for a single non-REM sleep bout recorded simultaneously from all areas. The gray-shaded area indicates the time corresponding to the traces in (A). Dotted lines indicate 100%. (**C**) FFT of power time course for sigma (left) and SWA (right) for individual mice (gray traces, *n =* 6) and for the average across mice (color + shading, means ± SEM). Open circles denote FFT peaks obtained from Gaussian fits. Vertical dotted lines indicate mean peak frequency ± 0.5 SD. (**D**) Mean peak values from (C) for sigma power and SWA for all brain areas and EEG recordings, analyzed as in Fig. 1D. RM ANOVA with factors “area” and “frequency”; area, *P* = 1.25 × 10^−6^; frequency, *P* = 6.88 × 10^−5^; post hoc paired *t* tests; EEG, *t* = 11.19, *P* = 9.93 × 10^−5^; SI, *t* = 17.88, *P* = 1.01 × 10^−5^; SII, *t* = 5.72, *P* = 0.0023; AC, *t* = 2.83, *P* = 0.037; mPFC, *t* = 2.02, *P* = 0.1; *P < 0.01, ***P < 0.001. SI and SII, primary and secondary somatosensory cortex; AC, auditory cortex; mPFC, medial prefrontal cortex; Ref, reference.

To assess whether 0.02-Hz oscillations were present in local cortical circuits, we performed multisite referential local field potential (LFP) recordings across four cortical areas in combination with polysomnography in sleeping head-fixed mice (*n =* 6). Under these recordings conditions, the three major vigilance states wake, non-REM, and REM sleep showed spectral profiles comparable to those of freely moving animals (fig. S8). The 0.02-Hz oscillation in sigma power of non-REM sleep was present in the simultaneously recorded EEG and LFP signals (Fig. 2, A and B), yet the latter showed that the amplitude of the oscillation depended on cortical area [*n =* 6 mice; repeated-measures (RM) analysis of variance (ANOVA) for factors “frequency” and “area”; *F*_1,5_ = 145.8, *P* = 6.88 × 10^−5^; *F*_4,20_ = 19.23, *P* = 1.25 × 10^−6^; Fig. 2C]. Primary (SI) and secondary (SII) somatosensory cortices showed a major 0.02-Hz peak in the sigma compared to the SWA power time course (*n =* 6; paired *t* test, *t* = 17.88, *P* = 1.01 × 10^−5^ for SI; *t* = 5.72, *P* = 0.0023 for SII; Fig. 2D), yet this peak was minor in auditory cortex (AC) and medial prefrontal cortex (mPFC) (*n =* 6; paired *t* test, *t* = 2.83, *P* = 0.037 for AC; *t* = 2.02, *P* = 0.1 for mPFC).

**Fig. 3:**
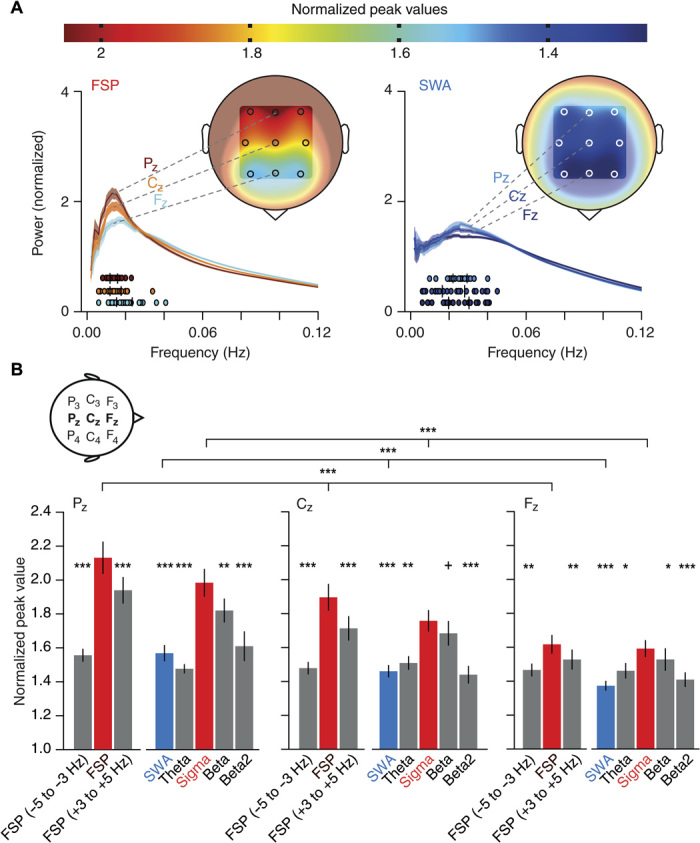
Regional cortical topology of the 0.02-Hz oscillation in humans. (**A**) Top: Color scale that indicates the mean normalized power values calculated from the average 0.02-Hz oscillation band (±0.5 SD around average peak values) during non-REM sleep. Bottom: The power spectral profiles for the FSP band (FSP ± 1 Hz, ~13 Hz, left) and the SWA band (right) averaged across subjects (color + shading, means ± SEM) displayed for representative midline electrodes (F_Z_, C_Z_, and P_Z_); analysis as in Fig. 1G. Coloring for power spectral profiles and each subject’s 0.02-Hz oscillation peak (filled circles underneath the power spectral profiles) corresponds to normalized peak values in the color scale. Insets show human head with an approximate topography of the mean normalized peak power values for all nine EEG electrodes (F_3_, F_Z_, F_4_, C_3_, C_Z_, C_4_, P_3_, P_Z_, and P_4_). (**B**) Mean (±SEM) normalized peak values for FSP band and adjacent frequency bands (FSP −5 to −3 Hz and FSP +3 to +5 Hz), as well as sigma power (10 to 15 Hz), SWA (0.5 to 4 Hz), theta (4 to 8 Hz), beta (16 to 20 Hz), and beta2 (20 to 24 Hz) bands separate for the three midline electrodes (F_Z_, C_Z_, and P_Z_); analysis as in Fig. 1H with data from the participants of the memory study (*n* = 24). Additional Friedman rank sum test between three midline electrodes for FSP band (*P* = 3.5 × 10^−8^), sigma (*P* = 6.35 × 10^−9^), and SWA (*P* = 2.8 × 10^−6^) (top three horizontal lines), with post hoc–paired comparisons along decreases from P_z_ to C_z_ as well as C_z_ to F_z_ separate for those three frequency bands (Wilcoxon signed-rank test, all *P*_s_ < 0.0036). For consistency with the core study analyses, which relied on nonparametric statistics, the same statistical tests were performed here. ^+^*P* < 0.1, *P < 0.05, **P < 0.01, ***P < 0.001 for Wilcoxon signed-rank test relative to FSP band (left bar groups) and relative to the sigma band (right bar groups).

The topography of 0.02-Hz oscillations in humans was assessed in an additional group of *n =* 24 subjects with full-night polysomnographic recordings (Fig. 3A and fig. S6). These data confirmed that the 0.02-Hz oscillation in sigma power was more pronounced during S2 than SWS. Furthermore, the 2-Hz band around the FSP was the strongest oscillatory component in these comparisons (fig. S6C). The 0.02-Hz oscillations showed a maximum over parietal derivations for power in both the sigma and the FSP band and declined toward anterior central and frontal areas. However, the relative dominance of the 0.02-Hz oscillation in the sigma and FSP bands over adjacent frequency bands and SWA persisted along the parietofrontal axis (Fig. 3B).

### The 0.02-Hz oscillation divides non-REM sleep into periods of high and low fragility to acoustic noise

**Fig. 4:**
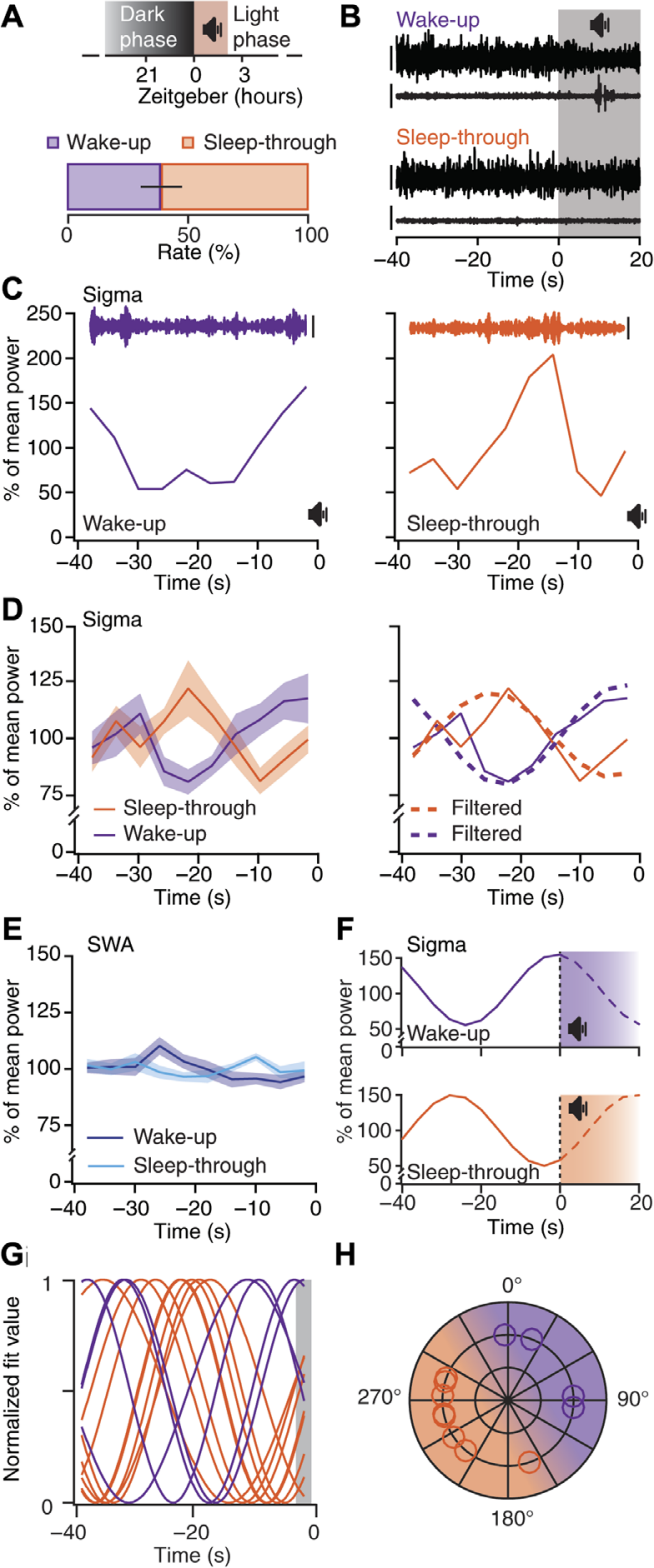
The 0.02-Hz oscillation imposes periods of high and low fragility to acoustic noise. (**A**) Top: Acoustic stimulation protocol. Bottom: Percentage (means ± SEM) of wake-up and sleep-through trials (*n =* 10 mice). (**B**) Representative EEG (ECoG) (upper trace)/EMG (lower trace) traces from wake-up and sleep-through trials. Gray-shaded area indicates period of noise exposure. Scale bars, 400 and 80 μV for EEG(ECoG)/EMG. (**C**) Time course of sigma power for the 40 s of non-REM sleep before noise onset for a wake-up (violet) and a sleep-through (orange) trial [same data as (B)]. Insets show corresponding band-pass–filtered (10 to 15 Hz) EEG (ECoG) traces. Scale bars, 200 μV. (**D**) Left: Means ± SEM sigma power time course for wake-up and sleep-through trials (*n =* 9 and 10, respectively; RM ANOVA for factors “time” and “behavioral outcome”, Greenhouse-Geisser–corrected, *P* < 0.0042). Right: Overlay of the traces from the left, once unfiltered (continuous line) and once band-pass–filtered (dotted lines) for the frequencies corresponding to 0.02-Hz oscillation peak ± 1 width (see Fig. 1C). (**E**) Means ± SEM SWA time course as in (D) [same statistics as in (D), *P* = 0.11]. (**F**) Projected time course of sigma power during noise exposure for wake-up and sleep-through trials. (**G**) Waveforms for average wake-up (*n =* 4) and sleep-through (*n =* 8) trials obtained through sinusoidal fits. (**H**) Polar representation of sigma power phases decoded from (G) (shaded area; 0°, peak), with shading of corresponding intervals for high (purple) and low (orange) responsiveness to stimulation.

If 0.02-Hz oscillations are relevant for sleep fragility and continuity, then they should be accompanied by a varying arousability of mice in response to external stimuli. We chose acoustic stimuli such that they lasted half a cycle of the 0.02-Hz oscillation (20 s). This long-duration noise would probe the propensity to arouse over the sustained periods of low and high sigma power and hence reveal whether these corresponded to states of distinct fragility. A white noise stimulus of 90-dB sound pressure level (SPL) yielded an arousal success rate of 38.7 ± 8.6% (*n =* 10), as assessed by polysomnography (Fig. 4A), and trials were post hoc–classified on the basis of ECoG (EEG)/EMG data in “wake-up” or “sleep-through” trials (Fig. 4B). Noise was played as soon as the mouse was in consolidated non-REM sleep (for ≥40 s) and at most once every 4 min, without knowledge of the oscillation phase. In a wake-up trial from a single mouse, sigma power was at its maximum before noise onset, such that noise exposure fell within a phase of declining power. In contrast, for a sleep-through trial of the same mouse, sigma power had just exited the trough, and noise was played within the phase of incrementing power (Fig. 4C). This phase difference between wake-up and sleep-through trials was robust when calculated across trials and mice (wake-up, *n =* 9 mice; sleep-through, *n =* 10 mice; RM ANOVA for factors “time” and “behavioral outcome”; *F*_4.78,81.27_ = 3.81, *P* < 0.0042, after Greenhouse-Geisser correction; Fig. 4D, left). Moreover, the time course corresponded to the 0.02-Hz oscillations during undisturbed sleep (Fig. 4D, right; see also Fig. 1), whereas SWA time course was indistinguishable between the wake-up and sleep-through trials (wake-up, *n =* 9 mice; sleep-through, *n =* 10 mice; RM ANOVA for factors “time” and “behavioral outcome”; *F*_4.84,82.20_ = 1.86, *P* = 0.11, after Greenhouse-Geisser correction; Fig. 4E). Therefore, as shown schematically in Fig. 4F, the 0.02-Hz sigma power oscillations seem to divide mouse non-REM sleep into alternating periods of successive high and low responsiveness to external stimuli. To test this hypothesis, we analyzed the phases of the 0.02-Hz oscillation before noise onset and found that values for the wake-up and sleep-through trials fell onto opposite halves in a polar plot of oscillation phases (Fig. 4, G and H). Therefore, wake-ups and sleep-throughs occur during declining and rising sigma power levels, respectively. As wake-ups took place either early or late during the 20-s noise exposure, we asked whether the declining sigma power phase could be further subdivided according to the occurrence of wake-ups. Sigma power was significantly lower for early (taking place within <8 s after noise onset) than for late (12 to 16 s after noise onset) wake-ups (early arousals, *n =* 6; late arousals, *n =* 9; RM ANOVA for factors “time” and “behavioral outcome”; *F*_4,52_ = 2.72, *P* = 0.04; fig. S9), suggesting a phase advancement for early over late arousals. The progression into the declining sigma power period thus reflects the entry into a period of sleep fragility. Last, we asked whether the total duration of the non-REM sleep before noise exposure affected responsiveness. Both wake-up and sleep-through trials were broadly distributed across the range of non-REM sleep bout durations in mice (fig. S10), ruling out bout duration as a determinant of behavioral outcome to noise exposure.

### The offline periods are coordinated with hippocampal ripples

We further characterized the 0.02-Hz oscillation by examining the timing of ripples (150 to 250 Hz) in the CA1 area of the hippocampus, which represent an established index for offline memory processing (*13*). Sigma power and ripple power were correlated such that ripple activity augmentations preceded sigma power rises by ~4 s (*n =* 6; fig. S11). Thus, hippocampal ripple activity was high during periods of increasing sigma power, during which mice maintained sleep while being exposed to noise. This suggests that more pronounced 0.02-Hz oscillations strengthen offline consolidation of hippocampus-dependent memory.

### The strength of the 0.02-Hz oscillation correlates with overnight consolidation of declarative memory in humans

**Fig. 5:**
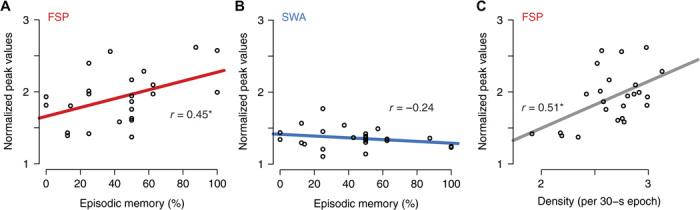
Sleep benefit in episodic memory correlates with the strength of the 0.02-Hz oscillation in the FSP band (FSP ± 1 Hz). (**A**) Correlation of episodic memory recall (that is, recall of objects in their spatiotemporal context) with normalized peak values of FSP band. Pearson’s *r* values are given in all panels (*P = 0.027). (**B**) Same for SWA band (*P* = 0.26). (**C**) Normalized power in FSP band was positively associated with the density of fast spindles (*P = 0.011). Analyses were performed on the average of all parietocentral EEG electrodes (C_3_, C_Z_, C_4_, P_3_, P_Z_, and P_4_) for the FSP band, and across frontal electrodes (F_3_, F_Z_, and F_4_) for the SWA band, as these sites correspond to the locations with the highest overall power in the respective bands.

To explore the role of 0.02-Hz oscillations in memory consolidation, we correlated the explicit postsleep recall on an episodic memory task (presented before sleep) in humans with oscillation peaks and conventional measures of spindle density (*14*). Recall correlated with the individual peak of 0.02-Hz oscillations in the fast spindle band during all-night non-REM sleep (*r* = 0.45, *P* = 0.027; *n =* 24; Fig. 5A), while correlation was absent for SWA (*r* = −0.24; Fig. 5B). The correlation episodic memory recall appeared to be most robust for the 0.02-Hz oscillation over centroparietal sites (C_4_, C_Z_, P_4_, and P_Z_; *P*_s_ < 0.043). The 0.02-Hz oscillation peak also correlated with the mean fast spindle density (*r* = 0.51, *P* = 0.011; Fig. 5C), but not with overall spindle count (*P* > 0.38). Spindle measures per se (such as spindle density and spindle count) in this analysis were not significantly correlated with episodic memory recall (all *P*_s_ > 0.15). These data are the first indications that the periodic clustering of spindle activity on a 50-s time scale is a critical determinant for offline memory consolidation.

### The online periods are coordinated with heart rate changes

**Fig. 6:**
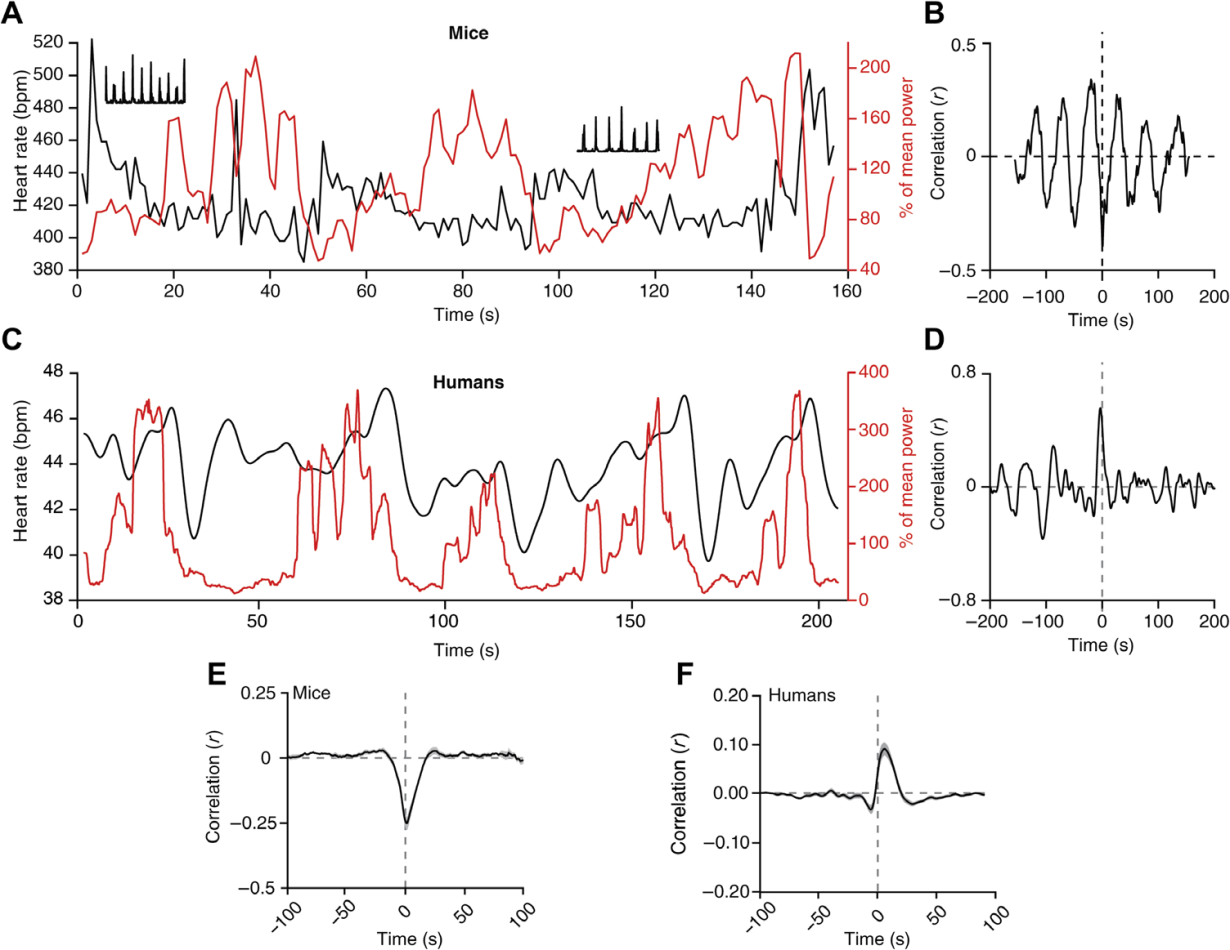
The 0.02-Hz oscillation aligns with heart rate changes in both mice and humans. (**A**) Representative non-REM sleep bout with simultaneous recording of sigma power (red trace) and heart rate [black trace; in beats per minute (bpm)]. Insets show 1-s period of corresponding raw data (squared) to illustrate R-wave detection in EMG traces. (**B**) Cross-correlogram between sigma power and heart rate for traces in (A). (**C**) Same as (A) for a single human subject. (**D**) Corresponding cross-correlogram as (B). (**E** to **F**) Mean cross-correlogram for mice (*n =* 12) (E) and humans (*n =* 27) (F). Shadowing represents means ± SEM.

To test our hypothesis that changes in heart rate accompany the period of fragility to external stimuli, we monitored heart rate along with non-REM sleep in both mice and humans. In mice, through measuring interbeat intervals from the nuchal EMG (fig. S12), we found that heart rate increased and fluctuated around elevated values when sigma power oscillations were declining (Fig. 6, A and B), yielding a cross-correlation function with a prominent negative peak at ~0 s (Fig. 6, B and E). In humans, heart rate alterations also correlated with sigma power, but with a clear time lag. Here, heart rate declined rapidly once sigma power had reached a peak and increased gradually during sigma power minima (Fig. 6, C and D), before subsequent sigma peaks by ~5 s (Fig. 6F). Thus, although with different phase relations that could be related to differences in the kinetics and mechanisms of neural coupling to the heart in both species, cardiovascular activity is coordinated with brain oscillations that mark arousability.

## DISCUSSION

Sleep has to reconcile the needs for continuity and fragility. Here, we uncovered a 0.02-Hz oscillation in mouse and human non-REM sleep with characteristics that qualify it as a hallmark for how sleep balances these conflicting needs. First, the 0.02-Hz oscillation is most prominent in a frequency band that contains neural rhythms associated with the gating of sensory information during sleep. Second, it is coordinated with an established physiological correlate of offline memory processing and with modulation of autonomic status. Third, the 0.02-Hz oscillation phase is linked to wake-up from sleep in mice and to the extent of overnight memory consolidation in humans. This dual behavioral relevance in two different species suggests that the 0.02-Hz oscillation provides a unitary temporal scale of mammalian non-REM sleep over which both beneficial effects and maintained reactivity to the environment are balanced.

### The 0.02-Hz oscillation in sigma power results from a periodic recurrence of sleep spindles

The 10- to 15-Hz frequency band analyzed here contains sleep spindles, a well-described sleep rhythm that is a thalamocortically generated and visually obvious hallmark of the non-REM sleep EEG in humans and carnivores between ~8 and 16 Hz (*7*, *15*). Mouse EEG traces show a more continuous and graded activity in the 9- to 18-Hz band that is best quantified through mean power levels (*11*). For species comparisons, we focused here on power dynamics in the 10- to 15-Hz band. The oscillatory pattern we found accords with the slow recurrence of discrete spindles over intervals of tens of seconds in humans and carnivores (*16*, *17*). Moreover, the much narrower individualized fast spindle band (around 13 Hz) oscillated most vigorously on a 0.02-Hz scale and correlated with the density of discrete spindles. Therefore, sleep spindles and fast spindles in particular are primary constituents of the 0.02-Hz oscillation. As adjacent frequency bands also show clear yet weaker 0.02-Hz oscillations, neural rhythm generators other than the thalamocortical spindle-generating circuits could contribute. In this context, it is noteworthy that the 8- to 13-Hz alpha band was recently associated with enhanced fragility of non-REM sleep in humans (*9*).

### The 0.02-Hz time scale is a fundamental property of mammalian non-REM sleep

Aside from shared spectral hallmarks and regulatory mechanisms, mouse and human non-REM sleep are strikingly different, in particular with respect to their architecture. In this study, we have now identified a temporal scale that is relevant for both S2 in humans and non-REM sleep in mice. We also have shown that mouse non-REM sleep shares several of the basic neural and autonomic characteristics of S2. These similarities will undoubtedly contribute to emerging questions on the specific benefits provided by S2 to sleep and in particular to sleep-dependent memory consolidation. We exemplify this here through demonstrating that the 0.02-Hz amplitude of fast spindles is a predictor of overnight declarative memory consolidation. Recent human research specifically linking S2 to strengthened hippocampal-cortical connectivity (*18*) and to procedural memory (*19*) is now open for reassessment in rodents in terms of novel temporal and spatial aspects of spindle organization.

The 0.02-Hz oscillation likely acts to provide an organizational time scale for non-REM sleep in other mammalian species. Carnivores, such as cats and ferrets, show periodically recurring spindle events at intervals of 10 to 40 s (*16*). Slow periodicities occur in brainstem arousal systems in sleeping rats (*20*). Beyond mammals, the Australian reptile *Pogona vitticeps* sleeps in alternating low (<4 Hz)– and high-frequency (10 to 30 Hz)–dominated states in cycles of 60 to 80 s (*21*).

The infraslow frequency of 0.02 Hz is strikingly similar to the periodicity found for cycling blood oxygen level–dependent imaging signals observed in brain subnetworks during rest (*22*) and non-REM sleep (*23*) that are conserved across rodents, monkeys, and humans (*24*) and that result from varying brain integration during sleep (*25*). Although the link between infraslow periodicities in electrophysiological and functional magnetic resonance imaging signals remains to be established, the shared oscillation frequency suggests that it represents an evolutionarily conserved time frame over which neural and hemodynamic sleep rhythms are coordinated.

### The 0.02-Hz oscillation renders human sleep S2 a functionally unique sleep stage

The predominance of 0.02-Hz oscillations for fast spindles in S2 of human non-REM sleep functionally sets S2 apart from SWS. Over a 50-s time scale, an S2-specific spindle amassment in parietal areas yields a qualitatively different spatiotemporal spindle pattern than in SWS, where cortically driven spindle grouping predominates (*8*). It remains to be determined how these diverse organizational hierarchies contribute to the differential alignment of fast and slow spindles with slow waves (*12*). To what extent the 0.02-Hz oscillation will be important for observed differences in local versus global recurrence of spindles during S2 and SWS (*26*), as well as for proposed frameworks on active systems consolidation (*27*), remains an additional area of future investigation.

### The online period of the 0.02-Hz oscillation facilitates wake-up in response to acoustic stimuli

Cortical responses to acoustic stimuli show an enhanced late inhibitory component of the evoked sensory responses during spindles (*6*, *28*), which is a neural correlate for disrupted cortical processing. The fragility period of the 0.02-Hz oscillation, corresponding to low spindle occurrence, could thus be accompanied by a suppression of these inhibitory components. However, the transition to full-blown awakening additionally requires an activation of brainstem arousal systems, such as the noradrenergic locus coeruleus that effectively arouses the thalamocortical system (*29*) and discharges phasically during alerting stimuli (*30*). Periodicities in sleep’s fragility to acoustic stimuli could be modulated through periodic patterns in excitability of this and/or additional subcortical arousal-promoting systems, which so far have not been investigated with respect to infraslow rhythms in activity during non-REM sleep (*20*).

Although a protective function of sleep spindles for arousals is well established, the role of the 0.02-Hz oscillation for arousability in humans will need to be ascertained to more comprehensively address the parallels between human sleep S2 and mouse non-REM sleep reported here. However, we caution here against a simple transfer of approaches between species. Not only do mice and humans differ in terms of subcortical and cortical mechanisms of sensory processing; stimulus attributes such as frequency composition also have different ecological valence (*31*). In humans, exposure to sounds mimicking those found in everyday life was previously used to assess resilience to sleep disruption and therefore seems suitable to address the role of the 0.02-Hz oscillation for sleep fragility in humans (*9*, *32*).

### The offline period of the 0.02-Hz oscillation promotes memory consolidation

The observed offline periods with reduced responsiveness to external noise might favor internal memory processing, as they coincide with enhanced ripple power, a sign for memory replay of recently experienced episodes (*13*). Human fast spindles predominate in sensorimotor areas and augment following learning (*33*) together with hippocampal ripples (*34*–*36*), a phenomenon that is crucial for memory consolidation (*1*, *37*). Our findings reveal the alignment of ripples and spindles within 25-s intervals that concur during periods of low fragility to noise. These data support the idea of a minimally required unit of uninterrupted sleep and provides a compelling explanation why optogenetically fragmenting non-REM sleep to periods shorter than 30 to 60 s disrupts memory consolidation (*38*). Corroborating the link to memory consolidation, we present the first evidence in humans that more pronounced 0.02-Hz oscillations in the spindle band correlated with enhanced hippocampus-dependent episodic memory after sleep. This further substantiates the idea that the temporal grouping of spindles, rather than their overall occurrence, is central to sleep-dependent memory consolidation.

### Alternative roles of the 0.02-Hz oscillation in non-REM sleep

Several facets of the 0.02-Hz oscillation support a role in subdividing sleep into fragility and offline periods, yet it undoubtedly serves roles that could complement or add to the ones presented here. These additional roles could include promotion of oscillatory signaling in signaling pathways in neurons and astrocytes, with implications for sleep-dependent gene transcription and synapse function. More generally, slow metabolic or energetic processes that result from, or contribute to, modified neuronal excitability during sleep (*39*) could evolve over infraslow time scales. Notably, oscillations in the 0.02-Hz range have also been reported in the EEG alpha and theta band activity during waking rest periods in humans (*40*). Furthermore, infraslow oscillations were observed in a broader frequency range (0.01 to 0.1 Hz) during a somatosensory detection task carried out in fully awake subjects (*41*), raising the possibility that neural variations on a 50-s time scale may be common to several vigilance states.

### Infraslow periodicities observed in clinical settings

Important clinical clues to infraslow periodicities in sleep fragility come from the “cyclic alternating pattern” (CAP) that is prominent in sleep disorder patients, consisting of visually identifiable alternations in EEG synchrony over periods of 10 to 60 s (*42*). Similar to the 0.02-Hz oscillation, these are coordinated with autonomic parameters and signs of elevated arousability, such as body movements. However, unlike the 0.02-Hz oscillation, the CAP occurs throughout all non-REM sleep stages with wide variations in its spectral composition. Infra-slow periodicities on a broader frequency range (0.01–0.1 Hz) have also been observed in the occurrence of epileptic seizures in humans (*43*), and hippocampal interneuron discharges in sleeping rats (*44*).

## CONCLUSIONS

In conclusion, the 0.02-Hz infraslow oscillation reflects sleep’s arbitration between maintaining readiness for arousal and continuity for offline processing. The oscillation provides a supraordinate temporal framework that, as we show here, controls sleep’s alternation between fragility and continuity, and which might likewise explain previously established links between sleep EEG rhythms, cardiac activity, hemodynamic fluctuations, and offline memory consolidation mechanisms (*22*, *23*, *45*) that occur on a 50-s time scale during sleep. Hypothalamic and brainstem circuits coordinating autonomic output with cortical state, possibly through diencephalic relays, are likely generators of the infraslow rhythm, which could affect cortical excitability (*46*, *47*). Therefore, we speculate that the 0.02-Hz infraslow oscillation reflects an inverse bottom-up oscillatory control between online and offline states, counterbalancing cortically driven faster sleep rhythms that organize brain activity in a top-down manner.

## MATERIALS AND METHODS

### Animal husbandry and experimental groups

Mice were housed in a temperature- and humidity-controlled environment with a 12-hour light/dark cycle (lights on from 9:00 a.m. to 9:00 p.m.). Food and water were administered ad libitum. Surgery for combined EEG (ECoG)/EMG electrode implantation was performed on a total of 26 5- to 7-week-old male C57BL/6J mice, bred in our colonies, as previously described by Wimmer *et al.* (*11*). For head-fixed conditions, eight C57BL/6J male mice of the same age were implanted for the EEG/EMG/LFP recordings. All experimental procedures complied with the Swiss National Institutional Guidelines on Animal Experimentation and were approved by the Swiss Cantonal Veterinary Office Committee for Animal Experimentation.

### Surgeries for polysomnography and LFP recordings in mice

For EEG (ECoG)/EMG surgeries, mice were anesthetized with isoflurane (1 to 2%, O_2_ and N_2_O mixture), and two gold-plated screws (1.1-mm diameter) (*48*) were gently inserted into the skull over the right hemisphere to obtain a frontoparietal derivation; four additional screws were inserted for implant stabilization. Two gold wires were inserted into the neck muscle for EMG recordings. A male-to-female connector was soldered to EEG (ECoG) and EMG electrodes, and the implant was covered with two-component epoxy glue (RelyX, 3M ESPE Dental Products; or G-CEM, GC Corporation) and dental cement (Paladur, Heraeus Kulzer GmbH). Paracetamol (2 mg/ml) was diluted into the drinking water for at least 10 days of recovery after the surgery, and an additional week of adaptation was given after the animals were tethered to a commutator (Dragonfly Inc.) via custom-made counterbalanced cables. Surgery for head-fixed LFP electrode implantation was performed under isoflurane anesthesia (1 to 2%, O_2_ and N_2_O mixture) on eight mice (*49*). Above the left hemisphere, small craniotomies were drilled (<0.5-mm diameter) to chronically implant LFP tungsten microelectrodes (FHC; 10 to 12 megohms) in the following areas of interest: AC (bregma posterior, −2.5 mm; lateral, 3.9 mm; surface depth, 1.0 mm), SI (bregma posterior, −1.7 mm; lateral, 3.0 mm; surface depth, 0.9 mm), SII (bregma posterior, −0.7 mm; lateral, 4.2 mm; surface depth, 1.0 mm), mPFC (prelimbic and infralimbic area: bregma anterior, +1.8 mm; lateral, 0.3 mm; surface depth, 1.85 mm), and CA1 (bregma posterior, −2.5 mm; lateral, 2 mm; surface depth, 1.3 mm). A silver wire (Harvard Apparatus) was positioned in contact with the bone above the cerebellum and used as a neutral reference to record referential LFP signals. Over the right hemisphere, a light metal implant was glued to the bone, and two EEG gold-plated wires were chronically implanted to record differential frontoparietal EEG signals similar to those of the freely moving animals. For EMG electrodes, two gold pellets were inserted into the neck. Carprofen (5 mg/kg subcutaneously) and paracetamol were provided during recovery from surgery. Mice were daily habituated by gradually increasing the amount of time in the head-fixed condition and by rewarding with sweet water after each session.

### Mouse polysomnographic and LFP recordings

EEG (ECoG)/EMG signals were recorded in freely moving mice, acquired and amplified using an Embla amplifier (gain 2000×), digitized at 2 kHz, and down-sampled to 200 Hz using Somnologica version 3.3.1 software (Embla System). The EEG (ECoG) and EMG traces were high-pass–filtered at 0.7 and 10 Hz, respectively. A 48-hour baseline sleep-wake recording under undisturbed conditions was obtained for every animal, and only the 100 min after light onset for the two consecutive days was used for further analysis to assess a data set homogeneous with respect to time of day. Recordings of LFPs were obtained from head-fixed mice habituated to sleep (fig. S8). The EEG (ECoG)/EMG signals allowed to assess the behavioral state during the sleep-wake cycle recordings and, together with LFP signals, were amplified (1000×) and acquired through Plexon Systems (16-channel Multiple Acquisition Processor system). More specifically, the signals were sampled at 1 kHz, high-pass–filtered at 0.8 Hz, and low-pass–filtered at 300 Hz. LFP electrode positions were labeled at the end of all recordings through electrocoagulation before transcardiac perfusion with 4% paraformaldehyde in 0.1 M phosphate buffer (under pentobarbital anesthesia, 60 mg/kg), through current injections (50 μA, 8 s), and post hoc compared to the stereotactic atlas after coronal slicing (100-μm sections) (fig. S8). Mice with an unprecise electrode localization were excluded from specific analyses. Head-fixed mice were not exposed to noise stimuli.

### Scoring of rodent polysomnographic and LFP data

All sessions involving freely moving animals were visually scored using a 4-s epoch resolution, and power spectra were determined as previously described by Wimmer *et al.* (*11*). Whenever an abnormal discharge was present or the behavioral state was unclear, the epoch was scored as an artifact corresponding to the closest behavioral state and was omitted for any spectral analysis. For any 4-s epoch to be included in the spectral time course, it had to be preceded and followed by another epoch belonging to the same behavioral state excluding artifacts. A vigilance state file and a spectral file (FFT, 0.75 to 90 Hz with 0.25-Hz steps) were exported from Somnologica for every 4-s epoch and for every recording session. Under the head-fixed condition, scoring was based on combined EEG/EMG/LFP data and involved the selection of consolidated non-REM sleep bouts ≥45 s, excluding transitional periods to REM sleep or waking. Power spectra were calculated with a 4-s window resolution. Scoring of EEG/LFP/EMG signals was performed using Igor Pro version 6.3 (WaveMetrics Inc.) customized semiautomated routines.

### Human subjects and sleep recordings

Human data obtained from 27 healthy men (22.5 ± 0.49 years of age; range, 18 to 28 years of age) who participated in a previous pharmacological study included overnight polysomnographic and electrocardiographic (ECG) recordings (*50*) (further referred to as “core study”) (the core analyses are presented in Figs. 1 and 6, and figs. S3, S4, S6A, and S7). Data for Figs. 3 and 5, and fig. S6 (B and C) were obtained from a sample that included 14 subjects taking part in a memory study (*14*) that was extended by 10 more subjects (*n =* 24, further referred to as “memory study”). The memory study also included standard polysomnographic full-night EEG recordings with a higher density of electrode sites.

All subjects had a regular sleep-wake pattern, did not take any medications at the time of the experiments, and were nonsmokers. Acute and chronic illness was excluded by medical history, routine laboratory investigation, and additional physical examination in the core study. The subjects of the core study were synchronized by daily activities and nocturnal rest with a more fixed sleep schedule, whereas the subjects of the memory study were instructed to keep their regular sleep schedule. Memory tasks were timed according to their regular sleeping time. All subjects spent one adaptation night in the laboratory to habituate to the experimental setting. For the core study, only data from placebo nights were included in the analysis. For the memory study, only the sleep group subjects were included. All participants gave written informed consent before participating, and both studies were approved by the local ethics committee.

Polysomnographic recordings included EEG from C_3_ and C_4_ electrode sites (International 10–20 system; reference: linked electrodes at the mastoids, ground at F_pz_), EMG (*musculus mentalis*), and electrooculography (around the eyes), with the memory study data set using additional EEG sites (F_3_, F_Z_, F_4_, C_3_, C_Z_, C_4_, P_3_, P_Z_, and P_4_). Electrode impedances were kept below 5 kilohms. Signals were amplified (BrainAmp, Brain Products), digitized (sampling rate >200 Hz), and filtered (EEG and electrooculogram between 0.3 and 35 Hz and EMG between 10 and 100 Hz).

### Scoring of human EEG data and sleep EEG parameter analyses

Sleep stages were scored offline in 30-s epochs by an experienced scorer according to standard criteria (*51*). Further analysis of the core study was focused on the first 210 min starting with sleep onset of undisturbed sleep that was expected to contain long uninterrupted epochs rich in S2 as well as SWS and good cardiac recording quality. The analysis of the memory study used the entire sleep period. The proportion of stage 1, S2, SWS (the sum of stage 3 and stage 4), non-REM sleep (sum of S2 and SWS), REM sleep, wakefulness after sleep onset, movement time, and sleep latencies was determined. Sleep onset was defined with reference to lights off by the first occurrence of an S1 sleep epoch followed by S2 sleep. For simplicity, EEG analysis focused on C_3_ channel mainly used for sleep scoring in the core study, whereas analyses in the memory group used all nine recording sites. Data of subsequent analyses were down-sampled to 100 Hz to facilitate computation. Analysis was performed in MATLAB 2013b (MathWorks) using custom-made scripts, FieldTrip (www.ru.nl/neuroimaging/fieldtrip) (*52*), and for analysis of standard sleep parameters including spindle and SWA analysis using the SpiSOP tool (www.spisop.org). For the correlation of the standard sleep parameters with memory and with the strength of the 0.02-Hz oscillation, the average of all parietocentral EEG electrodes (C_3_, C_Z_, C_4_, P_3_, P_Z_, and P_4_) was taken for the spindle band analyses, and the average of the frontal electrodes (F_3_, F_Z_, and F_4_) was taken for the SWA band analysis, because these locations correspond to the sites with the highest overall power in the respective bands.

Briefly, power spectral analyses of non-REM sleep were calculated on consecutive artifact-free 5-s segments of non-REM sleep, which overlapped in time by 4 s along the entire recording period. Each segment was tapered by a single Hanning window before applying an FFT that resulted in interval power spectra with a frequency bin resolution of 0.2 Hz. Power spectra were then averaged across all segments (Welch’s method). Mean power density from the spectra was calculated in the frequency band for slow waves (0.5 to 4 Hz) and in the sigma band (10 to 15 Hz). Concrete fast spindles and slow waves during non-REM sleep were analyzed according to previously published algorithms (*12*, *54*). For each individual and channel, their densities (per 30-s epoch of non-REM sleep), counts, mean amplitudes, and lengths were calculated.

For the identification of slow waves, the signal in each channel during non-REM sleep epochs was filtered between 0.5 and 3.5 Hz (−3 dB roll-off) using a digital finite impulse response (FIR) filter (Butterworth, order of 4). Next, all intervals of time with consecutive positive-to-negative zero crossings were marked as putative slow waves if their durations corresponded to a frequency between 0.5 and 1.11 Hz (*53*), yet these were excluded in case their amplitude was >1000 μV (as these were considered artifacts) or when both negative and positive half-wave amplitudes lay between −15 and +10 μV. A slow wave was identified if its negative half-wave peak potential was lower than the mean negative half-wave peak of all putatively detected slow oscillations in the respective EEG channel, and also only if the amplitude of the positive half-wave peak was larger than the mean positive half-wave amplitude of all other putatively detected slow waves within this channel.

For the detection of fast spindles, the EEG signal was filtered with a band-pass around the individual FSP (see “Analysis of 0.02-Hz oscillations in humans”) with a ±1-Hz range (−3 dB cutoff). Then, using a sliding window with a size of 0.2 s, the root mean square (RMS) was computed, and the resulting signal was smoothed in the same window with a moving average. A spindle was detected when the smoothed RMS signal exceeded an individual amplitude threshold by 1.75 × SD of the filtered signal in this channel at least once and additionally exceeded a lower threshold of 1.5 × SD for 0.5 to 3 s. The crossings of the lower threshold marked the beginning and end of each spindle and quantified their length. Spindle amplitude was defined by the voltage difference between the largest trough and the largest peak. Spindles were excluded for amplitudes >200 μV.

### Analysis of 0.02-Hz oscillations in mice

The scheme in fig. S2 summarizes the analysis steps for undisturbed non-REM sleep in mice. From the scored data, all non-REM sleep bouts ≥24 epochs (each epoch corresponding to 4 s, thus ≥96 s of uninterrupted non-REM sleep) were selected in the first 100 min at ZT0 for two consecutive light phases, regardless of the amount of non-REM sleep (fig. S2A). The spectral files of all 4-s epoch across all non-REM sleep (red lines in hypnogram in fig. S2A) were then used to calculate an arithmetic mean FFT per mouse. In recordings from head-fixed sleeping mice, an average non-REM sleep spectral profile was calculated across the entire recording for each mouse (810 to 3210 s).

Figure S2B shows how the time course of spectral power was analyzed in 4-s bins for the following frequency bands: SWA (0.75 to 4 Hz), theta (6 to 10 Hz), sigma (10 to 15 Hz), and beta (16 to 20 Hz). To do this, the power values from the 4-s FFTs for each frequency band were normalized to the average non-REM sleep FFT calculated across all non-REM sleep (fig. S2A) and plotted against time, yielding the line graphs in Figs. 1 (A and B) and 6A, and figs. S1, S2B, and S12.

The spectral profiles of these power time courses of each non-REM sleep bout were obtained through calculating an FFT (fig. S2C) with Hamming window method, which revealed all power values in the infraslow frequency range (<0.125 Hz). The choice of a minimal bout length of 24 epochs, corresponding to ≥96 s, preserved at least two cycles of the 0.02-Hz oscillation detected here. A mean spectral profile was calculated for every mouse through averaging across the interpolated absolute FFTs obtained from each power profile of each non-REM sleep bout (fig. S2C, right).

Control analyses were carried out to ensure the robustness of peak detection (as shown in fig. S3). For the analysis of scale-free behavior, similar FFT calculations for a simulated scale-free power profile (1/*f*) with equal bout length distribution did not yield a peak unless a 0.02-Hz sine wave was added (fig. S3, G to J). Before FFT calculation, the average power value for each frequency band was subtracted from each non-REM sleep bout to prevent large power increases at extremely small frequencies; however, the 0.02-Hz peaks were also present without this offset. Autocorrelations were calculated for original and shuffled sigma power data of these long non-REM bouts for each mouse (fig. S4, A and B).

The mean FFT obtained per mouse was normalized to its own mean. Similarly, in EEG (ECoG)/LFP recordings, the FFTs over the power data mentioned above were obtained for each non-REM sleep bout using a Hamming window and means calculated as before (Fig. 2). Leaving out the Hamming window did not affect the result.

Last, to determine peak and SDs of the FFTs, we performed a Gaussian fit (one term). Peak location and SD values were obtained from the fitted curve and used to calculate the average peak value in the range (peak ± 0.5 × SD). These values were used to calculate average peak values in all other frequency bands (Fig. 1D). By choosing a mean that is determined not only by the single data point of the peak but also by the spread of values around 1 full SD, we take into account that the exact peaks might not be identical for all frequency bands.

The peak ± SD frequencies of the FFT for sigma power were also used to calculate a band-pass (FIR)–filtered trace of sigma power time course before noise onset (Fig. 4D) and to reconstruct the oscillations in Fig. 4F. The peak ± SD frequencies of the FFT for sigma power were also used for the phase analysis in Fig. 4 (G and H). Here, the sigma power time courses in the prestimulus period were fit to a sinus function with a frequency constrained by these limits. The phase of these sinusoids was read for the wake-up and sleep-through trials in which both a peak and a trough lay within the fitted period. This was the case for 4 of 9 wake-up and 8 of 10 sleep-through sigma power time courses.

In noise exposure experiments, the time courses of sigma power and SWA in the 40-s prestimulus period were averaged across animals after sorting the trials on the basis of their outcome (wake-up or sleep-through trials). For any successful trial, the values in both sigma and SWA frequency bands were expressed in percentage with respect to the average non-REM sleep power spectrum in the 40-s prestimulus period (Fig. 4, C and E). Because of this normalization, 0.02-Hz oscillations had smaller amplitudes than those in Fig. 1. A total of *n =* 1 of 10 animals did not wake up in any of the noise exposures and did not contribute to the power calculations (Fig. 4, D and E). Later analysis showed that, in this mouse, only two noise exposures were successful, and both of these fell onto the rising phase of the sigma power oscillation. Analysis of phase was carried out with phase convention peak of 0° and trough of 180°, as described above.

Ripple activity time course was quantified from the LFP recording in the CA1 area. The signal was first filtered between 150 and 250 Hz and then squared. The values were averaged in a 4-s bin, and a cross-correlation was performed against the corresponding sigma power from channel SI (resampled at 1 Hz) using the ripple activity trace as “source wave.”

### Analysis of 0.02-Hz oscillations in humans

The scheme in fig. S5 summarizes the analysis steps for human non-REM sleep. An analysis similar to that in mice was performed on consecutive 30-s intervals of non-REM sleep EEG (further referred to as bouts) of the first 210 min of sleep and free of artifacts or movement arousals for the core study data set (fig. S5A) and was extended to the full-night sleep in the memory group data set (Figs. 3 and 5). The analysis differed from that in mice to account for dissimilarities in sleep patterns of humans (for example, longer bouts). For each EEG signal of a bout, the power spectra were calculated every 0.1 s in the 0.5- to 24-Hz range in steps of 0.2 Hz with a continuous wavelet transform using Morlet wavelets with length of four cycles. At every time point, the average power in the bands was calculated in frequency bands equivalent to mice: SWA (0.5 to 4 Hz), theta (4 to 8 Hz), sigma (10 to 15 Hz), individual beta (16 to 20 Hz), and beta2 (20 to 24 Hz). This resulted in a detailed power time course for each respective frequency band (fig. S5B).

After visual confirmation that the power time course in the sigma and SWA bands corresponded to real spindle and SWA activity, the temporal resolution was reduced to highlight activity changes in the infraslow periodicity. Therefore, a symmetric moving average was applied in a 4-s time window to match the resolution of the mouse data. As in mice, the average absolute values of the non-REM sleep spectral composition were used for normalization by setting them to 100% (Fig. 1, E and F). The first 100 min of concatenated non-REM sleep was used for this normalization to account for interindividual differences in the amount of non-REM sleep and to match the time interval used for the analysis in mice [fig. S5, A (bottom) and C].

To establish the spectral profile of these power time courses, the spectra of the power time courses for each frequency band were obtained for all non-REM sleep bouts lasting ≥120 s (≥4 epochs) (fig. S5A, bottom). For this, a Morlet wavelet analysis was performed in each interval on the previously calculated power time courses for respective frequency bands that aimed for a frequency resolution of 0.001 Hz in the range 0.001 to 0.12 Hz with time steps of 0.5 s. To obtain one power spectrum for each bout, we then averaged the resulting signal across time steps along the duration of the bout (fig. S5D).

Last, the spectral profile of the 0.02-Hz oscillation of all frequency bands for all non-REM sleep was calculated by averaging the spectral values across all the non-REM sleep bouts of the subject, weighted by their duration. As in mice, the spectral profile obtained per subject was normalized to its own mean. To determine peak and SDs of each power spectrum, a Gaussian fit (three terms) was performed on the normalized power from single subjects. The peak location and SD values were obtained from the fitted curve and used to calculate the average peak value in the range (peak ± 0.5 × SD) (Fig. 1, G and H). Accounting for the larger variability in individual peak values between human subjects and frequency bands as compared to mice, the respective range of each frequency band was used to approximate the highest possible average peak values. Using the range from the sigma band for averaging of peak values in all frequency bands essentially yielded the same results reported here.

To address specificity of the 0.02-Hz oscillation to spindles, we repeated the analysis for a frequency band tailored to the individual spindle band of each subject. Thus, for each subject, the frequency band was centered to its FSP that was determined according to previously described standard methods (*12*, *54*), and that is specified here briefly. Power spectra containing the sigma band (8 to 18 Hz) were calculated in the same way as reported above but using consecutive artifact-free 10-s intervals of non-REM sleep, which overlapped in time by 5 s with a frequency resolution of 0.1 Hz. The FSP was visually identified for each subject from the individual power spectra of non-REM sleep epochs as power maxima within the sigma band (*12*). Although slow spindles contribute to the sigma band, they were not considered because of their prevalence mainly during SWS and tight temporal association with fast spindles (*12*).

Control analyses for humans in figs. S3 and S4 including using minimal bout lengths of double (≥240 s; fig. S3, D to F) or triple the length (≥360 s), changing Morlet wavelet cycle length to seven cycles for better frequency resolution, skipping the normalization steps, or comparing the power fluctuations in the full spectrum (in smaller bands of 1 Hz from 1 to 24 Hz, instead of broader specific bands) essentially yielded the same results reported here. Autocorrelations were calculated as in mice for original and shuffled sigma power profile data of non-REM sleep. To more closely match the respective analyses in mice, the data were split in all possible continuous 240-s segments and down-sampled to 1 Hz (this analysis is referred to as 240-s bouts; see fig. S4, C and D).

### White noise exposure during polysomnographic recordings in sleeping mice

A subset of 10 mice implanted for polysomnography was habituated to the experimental noise stimulus during the period of tethering after surgery through playing noises six to eight times per light phase. During the experimental trials, animals were exposed, four at a time, to white noise pulses of 90-dB SPL lasting 20 s, generated through custom-written LabVIEW procedure (National Instruments Corporation). The duration of the noise was chosen such that it covered half a cycle of the 0.02-Hz oscillation and because mice woke up or slept through it at comparable rates. The arousal success rate was defined as the fraction of wake-up trials within all accepted trials. In a preliminary experimental series, a 20-s pulse at 80 dB was found to lead to an insufficient number of wake-up trials, with the arousal success rate <30%, whereas a 4-s pulse at 100 dB led to arousals in more than 90% of the cases. Noise was played randomly in the first 100 min at ZT0, but the following conditions had to be fulfilled: At least one of the four mice was in non-REM sleep for ≥40 s, as assessed through online monitoring of EEG (ECoG)/EMG traces, and the previous noise had been played ≥4 min before. The experimenter was blind to the spectral composition of non-REM sleep during the noise exposure experiment, such that noises were played without knowledge of the sigma power phase. Wake-ups were identified on the basis of characteristic alterations of EEG/EMG signals, namely, the decrease in amplitude and increase in frequency for the EEG trace, combined with the detection of muscular activity from the EMG electrodes (Fig. 4B). The animals were exposed several times to the noise in each recording session. The 20-s pulse was played 14.0 ± 0.3 (minimally 9) times per mouse, of which 8.6 ± 0.4 (minimally two per mouse) trials were successful, meaning that the mouse did not wake up in the prestimulus period or in the first 4 s of noise exposure. EEG (ECoG)/EMG traces were scored blind with respect to noise exposure times.

### Study procedures and memory assessment in humans

In the core study, subjects arrived at the laboratory at 9:00 p.m. for experimental preparation, and sleep was allowed between 11:00 p.m. (lights off) and 7:00 a.m. Subjects underwent blood sampling via an intravenous forearm catheter, which was connected to a long thin tube and enabled blood collection from an adjacent room without disturbing the subject’s sleep and unnoticed by all subjects (*14*).

The memory study demonstrated a twofold better recall of episodic memory when task performance was followed by nocturnal sleep compared to postlearning daytime wakefulness (*14*). Here, we only used the subjects of the sleep group, for which the procedures were as follows: Encoding of the memory task took place in the evening (between ~8:00 p.m. and 11:00 p.m.). One hour after encoding, and in accordance with their usual sleep habits, participants went to bed (lights off) for an 8-hour sleep period in the laboratory with polysomnographic recordings. The retrieval phase started 1 hour after awakening. The episodic memory task described in detail by Weber *et al.* (*14*) required the encoding of faces (events) embedded in a spatial context (that is, different locations on a screen) and a temporal context (that is, different faces at the different locations were presented in two experimental episodes separated by a 1-hour interval). During encoding, participants remained unaware that the task was aimed at memory testing but were instructed to keep focused on the experimental episodes presented on the screen.

For recall testing during the retrieval phase after sleep, old and novel faces were presented, and the subjects had to indicate (by mouse clicks) whether a face was new or presented during one of the task episodes, and for the latter, whether it occurred in the first or second episode and at which location it occurred. Episodic memory, that is, “what-where-when” memory, was determined by the percentage of the faces that were correctly identified as occurring in one of the episodes (that is, “what”), and for which the participant also correctly indicated the episode (that is, “what-when”) and the location (that is, “what-where”) it occurred, minus the locations for which the participant had forgotten that they were occupied with any face in a final separate recall test (false “where-when” memory).

### Analysis of heart rate in mice and humans

The instantaneous heart rate was extracted from the nuchal EMG recording in freely moving or head-fixed mice and calculated from the ECG recordings in humans. In *n =* 12 mice (eight freely moving and four head-fixed), the heart rate was quantified through detection of R waves in the EMG trace filtered between 20 and 300 Hz. Reliability of this signal was confirmed through standard ECG recording in one mouse (fig. S12) (*55*). Peak or threshold detections were used, with equal results. In the latter case, threshold was set as the mean + 3.5 × SD of the EMG signal. We then calculated the interval between two successive peaks (R-R interval) for all consolidated non-REM sleep bouts ≥45 s. Occasionally, R waves were classified as aberrant because they were either below threshold or artifactual because of muscle twitches, which corresponded to 1.41% of all intervals. In freely moving animals, all non-REM sleep bouts ≥96 s were used. The number of non-REM sleep bouts per head-fixed animal included in the analysis ranged from 10 to 42 bouts (mean number of bouts, 22.3 ± 7.2). The R-R intervals were then binned (1 s) and converted into beats per minute. In humans, the heart rate was determined across artifact-free consecutive non-REM sleep intervals on the basis of R-R intervals. R waves were detected by first filtering the ECG signal with a high-pass filter of 20 Hz [infinite impulse response (IIR); designed for a stopband of 15 Hz with −100-dB attenuation, with two filter passings and no time shift] and then applying a low-pass filter at 45 Hz (IIR; filter order of 4, with two filter passings and no time shift). To obtain a clear signal amplitude envelope describing the R wave, we calculated the absolute values of the Hilbert transform of the signal. Then, the signal was down-sampled to 100 Hz to facilitate computation. R-wave peaks were automatically identified as maxima in the envelope signal if they were at least 0.2 s apart (minimal heart refractory period) and reached above a threshold of 2 SDs from overall envelope signal values. This method was visually confirmed in each subject to validate correct R-wave peak detection in all epochs. Instantaneous heart rate at every R-wave peak was then determined by duration between consecutive R-R intervals.

For cross-correlating time courses of heart rate (in beats per minute) and sigma power in mice, the sigma power trace was interpolated to match the 1-Hz sampling rate of the heart rate trace. In humans, it was smoothed in a 4-s moving symmetric time window, and both sigma power and heart rate traces were resampled at 100 Hz by interpolation. The 120-s intervals were *z*-transformed (by subtracting the mean and dividing by the SD). In both mice and humans, the cross-correlation was performed using the heart rate signal as source wave. In both species, the cross-correlograms were first averaged within and then across subjects. In humans, all of the above procedures were repeated separately for S2 and SWS epochs instead of non-REM sleep epochs but reported solely for the oscillation peak analysis.

### Experimental design and statistics

Group size in mice matched the minimum required to obtain a statistical power of 0.8. Power analyses were carried out on the basis of effect sizes obtained from preliminary data sets. Group size in humans was chosen to obtain a statistical power of 0.95 for similar effect sizes, as observed in mice. Statistical power was calculated using G*Power version 3.1.9.2. Data normality was tested using the Shapiro-Wilk *W* test, and parametric or nonparametric statistical tests were chosen accordingly. The Wilcoxon signed-rank test was used for nonparametric matched pair comparisons, whereas paired two-tailed Student’s *t* tests (referred to as *t* test in text and legends) were used as parametric statistical tests. RM ANOVA was used as parametric statistical test to study the within-subjects effect of behavioral outcome and/or the between-subjects effect. To assess equality of variances for the RM ANOVA, we calculated the Mauchly’s test of sphericity. Whenever equality of variances was rejected, the univariate adjusted Greenhouse-Geisser correction was applied. Friedman rank sum test was used as a nonparametric test to study within-subjects effect in case parametric model parameters of an ANOVA violated assumptions of normality. Statistical tests were calculated using JMP version 10.0.0 (SAS Institute Inc.), Igor Pro, and the R programming language (2.15.0, R Core Team) [The R Development Core Team, The R Foundation for Statistical Computing (www.r-project.org/foundation), 2007]. *P* < 0.05 was considered statistically significant. If not mentioned otherwise, *P* values were reported uncorrected for multiple comparisons, as taking these into account did not alter the main results. For the purpose of comparison between mice and humans, all data in bar graphs are presented as means ± SEM, even if not normally distributed (Fig. 1, D, H, and J). All indications of *n* refer to either mice or humans. Time course graphs, as well as data in the main text, are presented as means ± SEM.

## Supplementary Material

http://advances.sciencemag.org/cgi/content/full/3/2/e1602026/DC1

## SUPPLEMENTARY MATERIALS

Supplementary material for this article is available at http://advances.sciencemag.org/cgi/content/full/3/2/e1602026/DC1 fig. S1. The 0.02-Hz oscillation is prominent for sigma power throughout both short and long non-REM sleep bouts in mice. fig. S2. Scheme of analysis for 0.02-Hz oscillations in mice. fig. S3. The 0.02-Hz oscillation is robust against the choice of non-REM sleep bout length for analysis and does not result from an 1/*f* power dependence. fig. S4. The sigma power dynamics in both mice and humans show a periodicity on a 0.02-Hz time scale, as assessed through autocorrelations. fig. S5. Scheme of analysis for 0.02-Hz oscillations in humans. fig. S6. Sleep parameters for the participants of the studies in humans and predominance of 0.02-Hz oscillations in S2 sleep. fig. S7. The 0.02-Hz oscillation is prominent for sigma power throughout early non-REM sleep in humans. fig. S8. Sleep in head-fixed animals reproduces the three major vigilance states and their spectral characteristics found in freely moving animals. fig. S9. Acoustic stimuli causing early or late wake-ups fall onto late or early portions of the declining sigma power phase, respectively. fig. S10. Wake-up and sleep-through trials do not depend on previous sleep duration. fig. S11. Ripple power increases precede sigma power elevations. fig. S12. Nuchal EMG recordings faithfully detect the R-waves of the heartbeat in mice.
