# Erratum for the Research Article: “Coordinated infraslow neural and cardiac oscillations mark fragility and offline periods in mammalian sleep” by S. Lecci, L. M. J. Fernandez, F. D. Weber, R. Cardis, J.-Y. Chatton, J. Born, A. Lüthi

**DOI:** 10.1126/sciadv.aaq0565

**Published:** 2017-09-29

In the article “Coordinated infraslow neural and cardiac oscillations mark fragility and offline periods in mammalian sleep,” the sentence in the Discussion section wrongly read “However, unlike the 0.02-Hz oscillation, the CAP occurs throughout all sleep stages with wide variations in its spectral composition, occurrence of epileptic seizures in humans (*43*), and hippocampal interneuron discharges in sleeping rats (*44*).” The sentence was corrected to read “However, unlike the 0.02-Hz oscillation, the CAP occurs throughout all non-REM sleep stages with wide variations in its spectral composition. Infra-slow periodicities on a broader frequency range (0.01–0.1 Hz) have also been observed in the occurrence of epileptic seizures in humans (*43*), and hippocampal interneuron discharges in sleeping rats (*44*).“ The HTML and PDF versions of the paper were corrected 29 June 2017 and an erratum was published 29 September 2017.

