# Supplementary material for: Coordinated infraslow neural and cardiac oscillations mark fragility and offline periods in mammalian sleep
Source: Sci Adv. 2017 Feb 8;3(2):e1602026. doi: 10.1126/sciadv.1602026 (PMC5298853; doi:10.1126/sciadv.1602026)
Supplement: http://advances.sciencemag.org/cgi/content/full/3/2/e1602026/DC1 [file supp_3_2_e1602026__index.html]

Science Advances | Science Advances

## Supplementary Materials

**This PDF file includes:**

- fig. S1. The 0.02-Hz oscillation is prominent for sigma power throughout both short and long non-REM sleep bouts in mice.
- fig. S2. Scheme of analysis for 0.02-Hz oscillations in mice.
- fig. S3. The 0.02-Hz oscillation is robust against the choice of non-REM sleep bout length for analysis and does not result from an 1/*f* power dependence.
- fig. S4. The sigma power dynamics in both mice and humans show a periodicity on a 0.02-Hz time scale, as assessed through autocorrelations.
- fig. S5. Scheme of analysis for 0.02-Hz oscillations in humans.
- fig. S6. Sleep parameters for the participants of the studies in humans and predominance of 0.02-Hz oscillations in S2 sleep.
- fig. S7. The 0.02-Hz oscillation is prominent for sigma power throughout early non-REM sleep in humans.
- fig. S8. Sleep in head-fixed animals reproduces the three major vigilance states and their spectral characteristics found in freely moving animals.
- fig. S9. Acoustic stimuli causing early or late wake-ups fall onto late or early portions of the declining sigma power phase, respectively.
- fig. S10. Wake-up and sleep-through trials do not depend on previous sleep duration.
- fig. S11. Ripple power increases precede sigma power elevations.
- fig. S12. Nuchal EMG recordings faithfully detect the R-waves of the heartbeat in mice.

Download PDF

**Files in this Data Supplement:**

- Adobe PDF - 1602026\_SM.pdf
