# Supplementary material for: Coordinated infraslow neural and cardiac oscillations mark fragility and offline periods in mammalian sleep
Source: Sci Adv. 2017 Feb 8;3(2):e1602026. doi: 10.1126/sciadv.1602026 (PMC5298853; doi:10.1126/sciadv.1602026)
Supplement: http://advances.sciencemag.org/cgi/content/full/3/2/e1602026/DC1 [file 1602026_SM.pdf]

Science Advances

AAAS

advances.sciencemag.org/cgi/content/full/3/2/e1602026/DC1

Supplementary Materials for

Coordinated infraslow neural and cardiac oscillations mark fragility and offline periods in mammalian sleep

Sandro Lecci, Laura M. J. Fernandez, Frederik D. Weber, Romain Cardis, Jean-Yves Chatton, Jan Born, Anita Lüthi

Published 8 February 2017, *Sci. Adv.* **3**, e1602026 (2017)
DOI: 10.1126/sciadv.1602026

This PDF file includes:

- fig. S1. The 0.02-Hz oscillation is prominent for sigma power throughout both short and long non-REM sleep bouts in mice.
- fig. S2. Scheme of analysis for 0.02-Hz oscillations in mice.
- fig. S3. The 0.02-Hz oscillation is robust against the choice of non-REM sleep bout length for analysis and does not result from an 1/*f* power dependence.
- fig. S4. The sigma power dynamics in both mice and humans show a periodicity on a 0.02-Hz time scale, as assessed through autocorrelations.
- fig. S5. Scheme of analysis for 0.02-Hz oscillations in humans.
- fig. S6. Sleep parameters for the participants of the studies in humans and predominance of 0.02-Hz oscillations in S2 sleep.
- fig. S7. The 0.02-Hz oscillation is prominent for sigma power throughout early non-REM sleep in humans.
- fig. S8. Sleep in head-fixed animals reproduces the three major vigilance states and their spectral characteristics found in freely moving animals.
- fig. S9. Acoustic stimuli causing early or late wake-ups fall onto late or early portions of the declining sigma power phase, respectively.
- fig. S10. Wake-up and sleep-through trials do not depend on previous sleep duration.
- fig. S11. Ripple power increases precede sigma power elevations.
- fig. S12. Nuchal EMG recordings faithfully detect the R-waves of the heartbeat in mice.

SUPPLEMENTARY MATERIALS

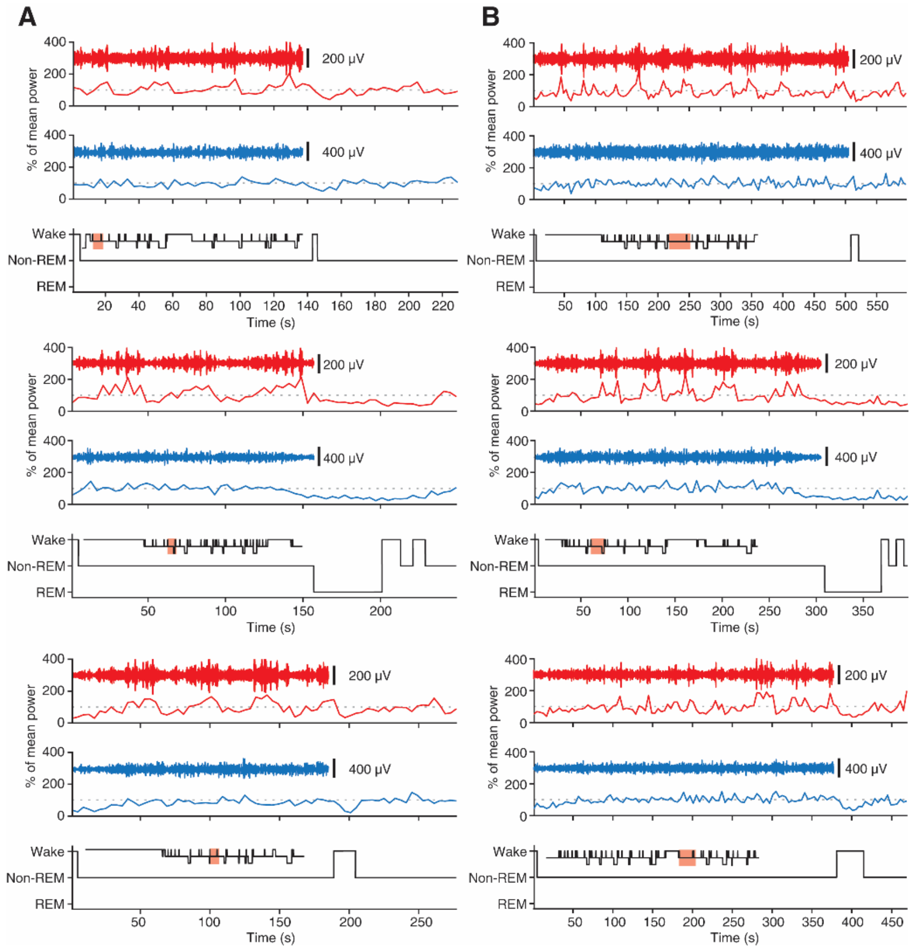

fig. S1. The 0.02-Hz oscillation is prominent for sigma power throughout both short and long non-REM sleep bouts in mice. (**A** and **B**) Power dynamics from both short (A) and long (B) non-REM sleep bouts from different mice, presented as in Fig. 1B. These mice are different from the one shown in Fig. 1. Insets show the hypnogram for the entire 100 min selected for the analysis, with red shadowing corresponding to the presented bout. Note the clear presence of 0.02-Hz oscillations in all bouts, irrespective of their duration and time of occurrence within the light phase.

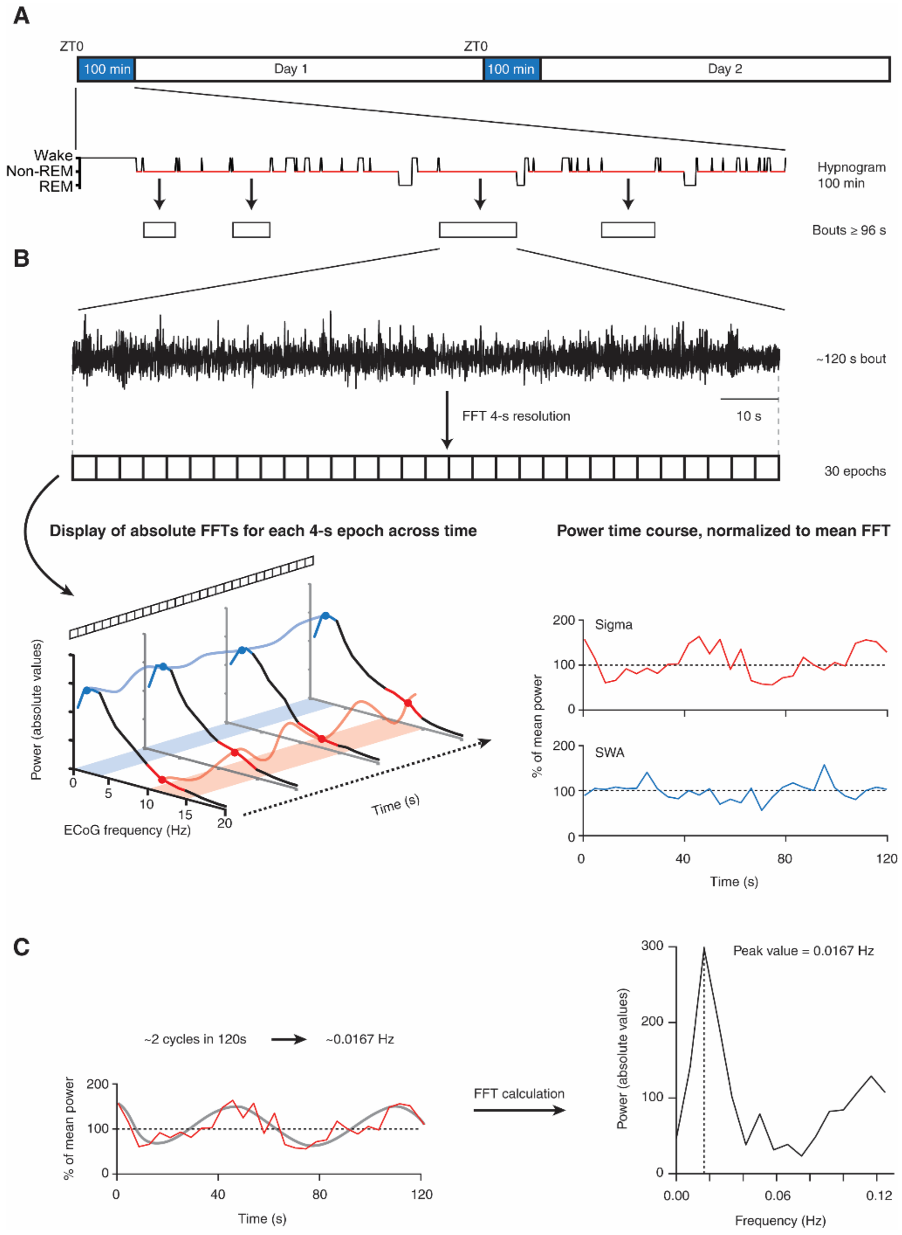

fig. S2. Scheme of analysis for 0.02-Hz oscillations in mice. See Materials and Methods, wherein each step is described in detail in a separate paragraph. (**A**) Selection of non-REM sleep bouts ≥96 s in the first 100 min of the two light phases. (**B**) Calculation of the power time course for the different frequency bands. In the left panel, 4 absolute FFTs out of a total of 30 epochs are displayed. Blue and red lines sketch a hypothetical time course of power values in the SWA and the sigma power range, respectively. In the right panel, the power time course across the entire bout, derived from the 30 FFTs of the original dataset, is illustrated.

(**C**) Calculation of the spectral profile of the power time course that results in the detection of the 0.02-Hz oscillation.

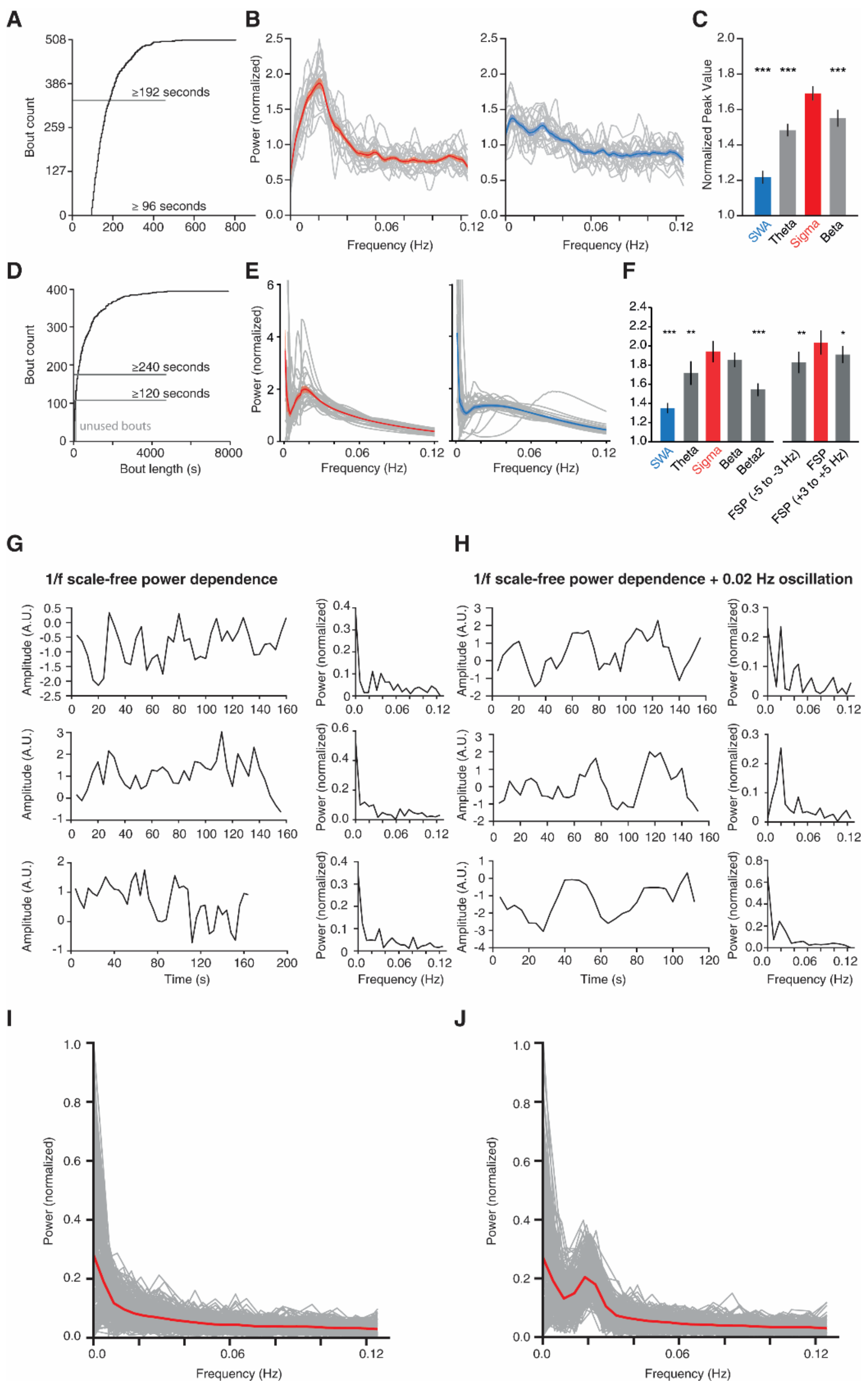

fig. S3. The 0.02-Hz oscillation is robust against the choice of non-REM sleep bout length for analysis and does not result from an 1/*f* power dependence. (**A**) Cumulative distribution of bout lengths ≥96 s across *n* = 18 animals for two consecutive light phases, with horizontal line indicating the value of 192 s, the threshold for longer bouts selected here. (**B** and **C**) Same analysis as in Fig. 1, C and D for these longer bouts. Note that a 0.02-Hz peak

was predominant in the dynamics of the sigma power compared to SWA. Statistics as in Fig. 1D, $P = 1.37 \times 10^{-9}$, ***$P < 0.001$. (**D** to **F**) Same analysis for human for bout lengths ≥ 240 s. All bouts are shown in the cumulative distribution. Statistics as in Fig. 1H, Left, $P = 1.417 \times 10^{-4}$, **$P < 0.01$, ***$P < 0.001$. Right, around the bands of fast spindle power peak (FSP) $P = 0.054$, *$P < 0.05$, **$P < 0.01$. (**G** and **H**) For comparison, individual traces obtained from simulated dataset with 1/f power dependence (*42*). In H, an additional 0.02-Hz oscillation with amplitude corresponding to the largest frequencies in the 1/f spectrum is superimposed, and their corresponding FFTs are shown. (**I** and **J**) Superimposed FFTs of >270 individual traces (gray) with length distribution corresponding to that obtained from our mouse dataset (mean 108.6 ± 8.1 s). A 0.02-Hz peak does not appear in the Fourier transform of such a dataset unless a 0.02-Hz oscillation is specifically added. Mean ± SEM shown in red ± shadow. A.U., arbitrary units.

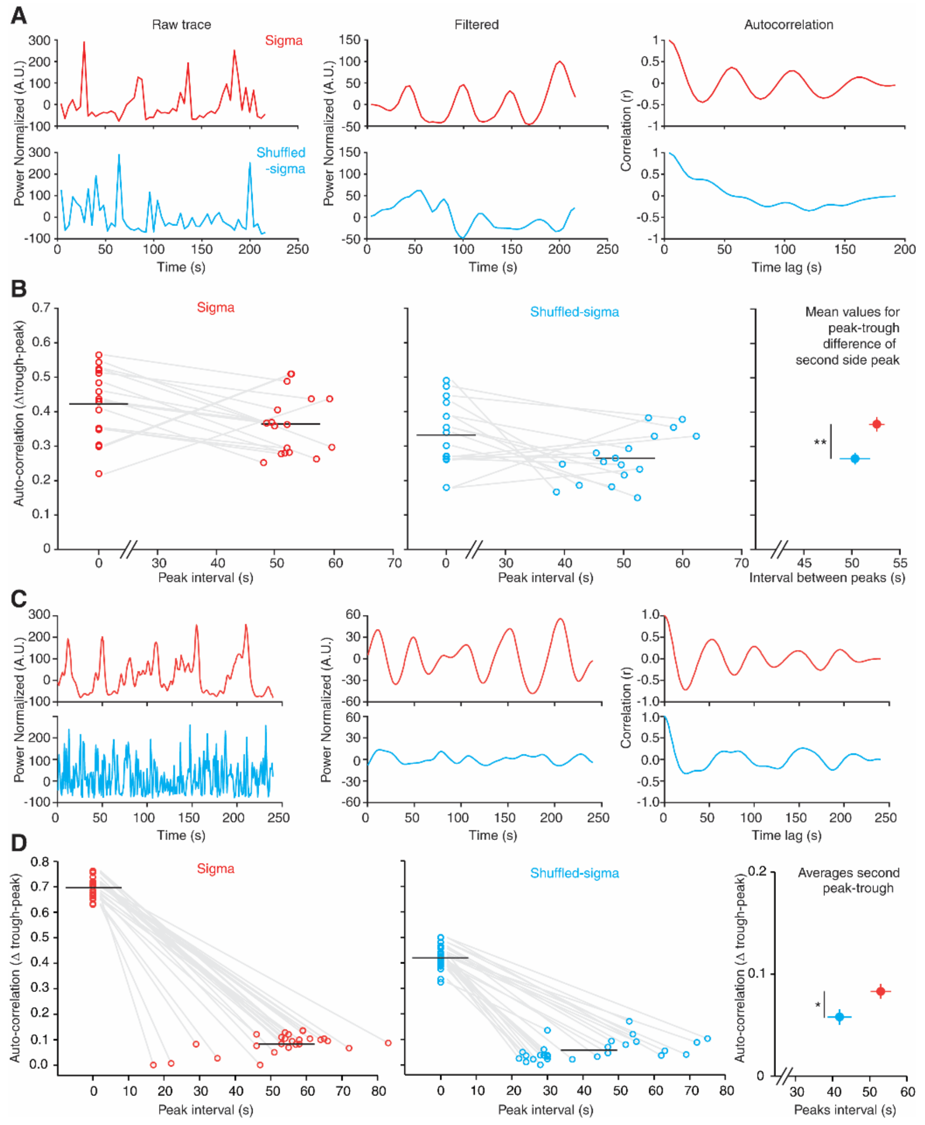

fig. S4. The sigma power dynamics in both mice and humans show a periodicity on a 0.02-Hz time scale, as assessed through autocorrelations. (**A**) Single trace from one non-REM sleep bout in mouse as recorded originally (red, left), after filtering between 0.015 and 0.025 Hz (red, middle) and after calculating the autocorrelation (red, right). Corresponding green traces show the same for randomly shuffled sigma power data (light blue). The original and shuffled data also differed with respect to the variability of the time interval between the peaks (*F*-test, $F = 3.79$, $P = 0.011$). (**B**) Quantification of autocorrelation for $n = 18$ mice through peak-trough differences of the first and second side-peaks in non-REM sleep bouts lasting ≥192 s for original (red open circles) and shuffled data (light blue open circles). The

values for the first peak are set to the timepoint zero, whereas those of the second peak are positioned according to their intervals with respect to the first peak. Peak-trough differences were detected automatically through searching for maxima and minima. Horizontal black lines denote mean values. On the right, mean ± SEM values of the second side peak are given for original and shuffled data, with SEMs calculated for both amplitudes and time intervals from the first peak. $^{**}P = 0.0015$, paired *t* test. (**C** and **D**) Same as in A and B for $n = 27$ humans using 240-s non-REM sleep bouts. $^{*}P = 0.023$, Wilcoxon signed-rank test. Similar tests for SWA in mice and humans did not yield a periodicity that was significantly different from the shuffled data. A.U., arbitrary units.

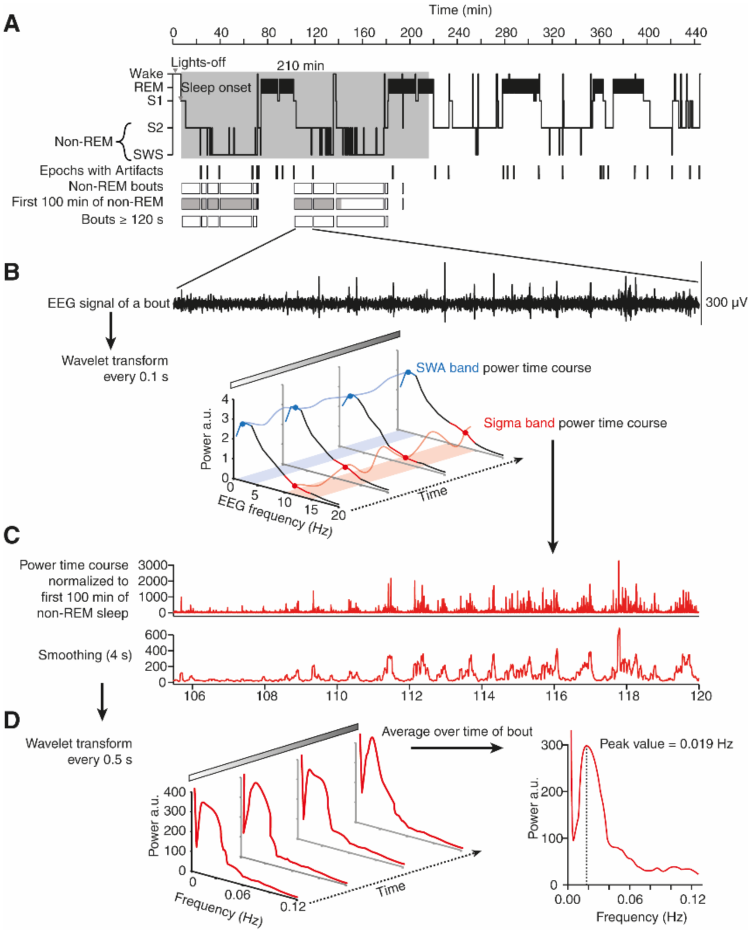

fig. S5. Scheme of analysis for 0.02-Hz oscillations in humans. See Materials and Methods, wherein each step is described in detail in a separate paragraph. (**A**) Selection of non-REM sleep bouts ≥120 s in the first half of a night (first 210 min of sleep, hypnogram period shaded gray) and the intervals used for normalization of subsequent power time course (first 100 min of non-REM sleep bouts, bout periods shaded gray). For the memory dataset the full-night ~8 hours of sleep were used for analysis. (**B**) Calculation of a hypothetical power time course for the different frequency bands for one bout. For illustration, four example power spectra are visually displayed. (**C**) Further normalization and smoothing of power time course, presented here for the sigma band. (**D**) Calculation of the spectral profile of the power time course that results in the detection of the 0.02-Hz oscillation. A.U., arbitrary units.

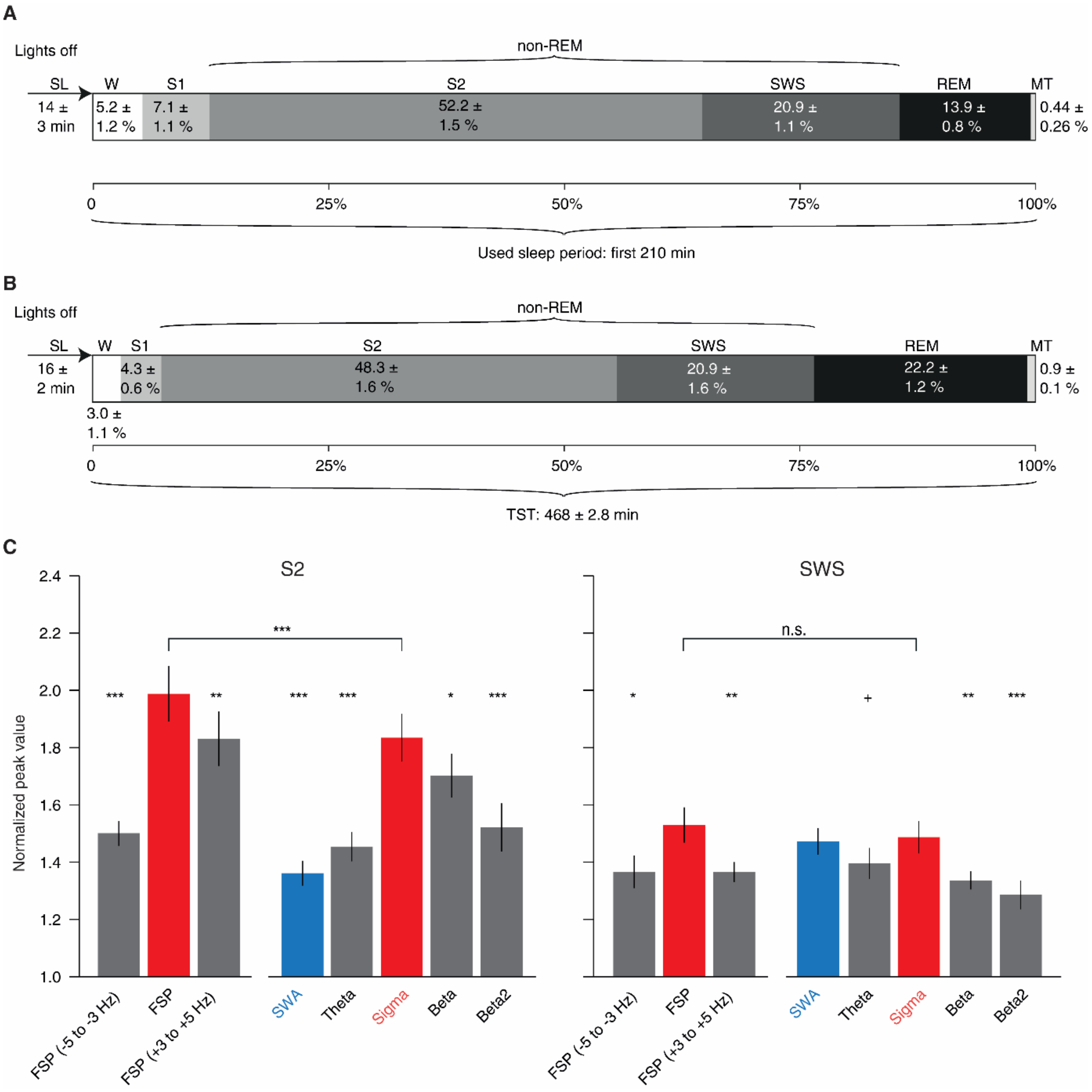

fig. S6. Sleep parameters for the participants of the studies in humans and predominance of 0.02-Hz oscillations in S2 sleep. (**A**) Sleep parameters for participants of the core study (*n* = 27), including sleep onset latency (SL), and percentages of wake after sleep onset (W), stage 1 (S1), S2, SWS, non-REM (S2 + SWS), REM sleep and movement time (MT). Parameters are given for the first 210 min of sleep time used in the presented analyses. (**B**) Same parameters for the participants of the memory study (*n* = 24). Parameters are given with reference to the total sleep time (TST) during the entire nocturnal sleep period. (**C**) Mean (± SEM) normalized power values calculated from the average 0.02-Hz oscillation

band (± 0.5 SD around average peak values) from the power spectral profiles of the power time course of the FSP band and adjacent frequency bands (FSP -5 to -3 Hz and FSP +3 to +5 Hz), as well as sigma (10–15 Hz), SWA (0.5–4 Hz), theta (4–8 Hz), beta (16–20 Hz) and beta2 (20–24 Hz) bands; data from the n = 24 subjects of the memory. Left, data from S2 sleep only. Right, data from SWS only. FSP band in S2 was the strongest component over all other frequency bands, including the sigma band (***$P = 4.75 \times 10^{-4}$ for Wilcoxon signed-rank test); FSP band was not stronger than sigma band in SWS (n.s. $P > 0.56$). The predominance of 0.02-Hz oscillations in the sigma band over the SWA band exclusively in S2 sleep (with diminished 0.02-Hz oscillations in those two bands in SWS) was confirmed by a RM ANOVA with significant interaction of the factors "frequency" (Sigma vs. SWA) and "sleep stage" (S2 vs. SWS) ($F_{(1,23)} = 21.99$, $P = 1.01 \times 10^{-4}$ for interaction effect; $F_{(1,23)} = 18.87$, $P = 2.39 \times 10^{-4}$ for main effect of frequency; $F_{(1,23)} = 4.29$, $P = 0.0497$ for main effect of sleep stage). Due to artifacts, one subject was excluded in the FSP band analysis on SWS data (n = 23). For consistency with the core study analyses which relied on nonparametric statistics, the same statistical tests were performed here. +$P < 0.1$, *$P < 0.05$, **$P < 0.01$, ***$P < 0.001$ for Wilcoxon signed-rank tests with respect to FSP band (left bar groups) or sigma band (right bar groups). Analyses as in Fig. 1, H and J.

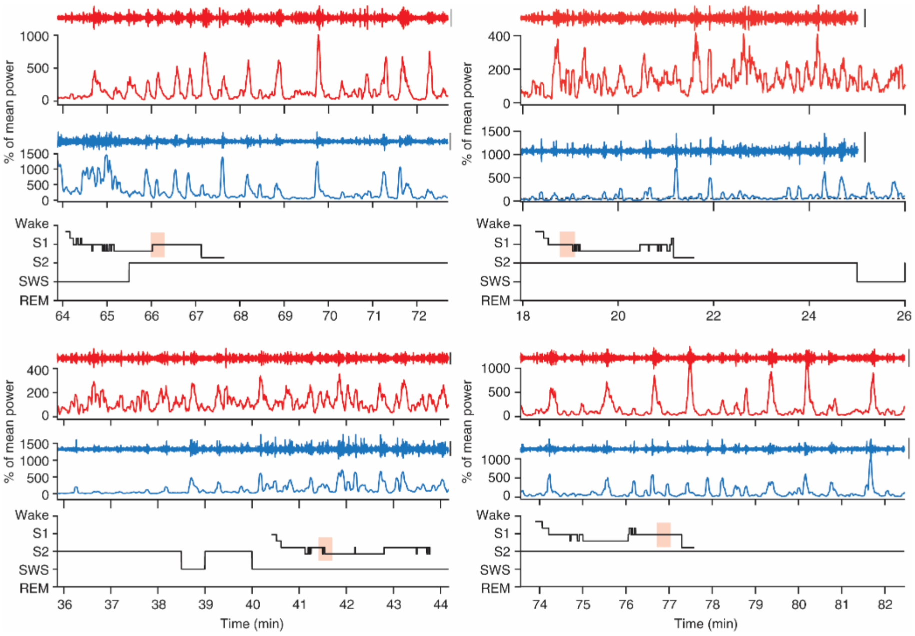

fig. S7. The 0.02-Hz oscillation is prominent for sigma power throughout early non-REM sleep in humans. Power time courses of four different human subjects presented as in Fig. 1F. Hypnograms from the first 90 to 120 min, corresponding to the first sleep cycle, are shown as insets with red shadowing indicating the presented segments. Scalebars, 40 µV for red trace, 200 µV for blue trace.

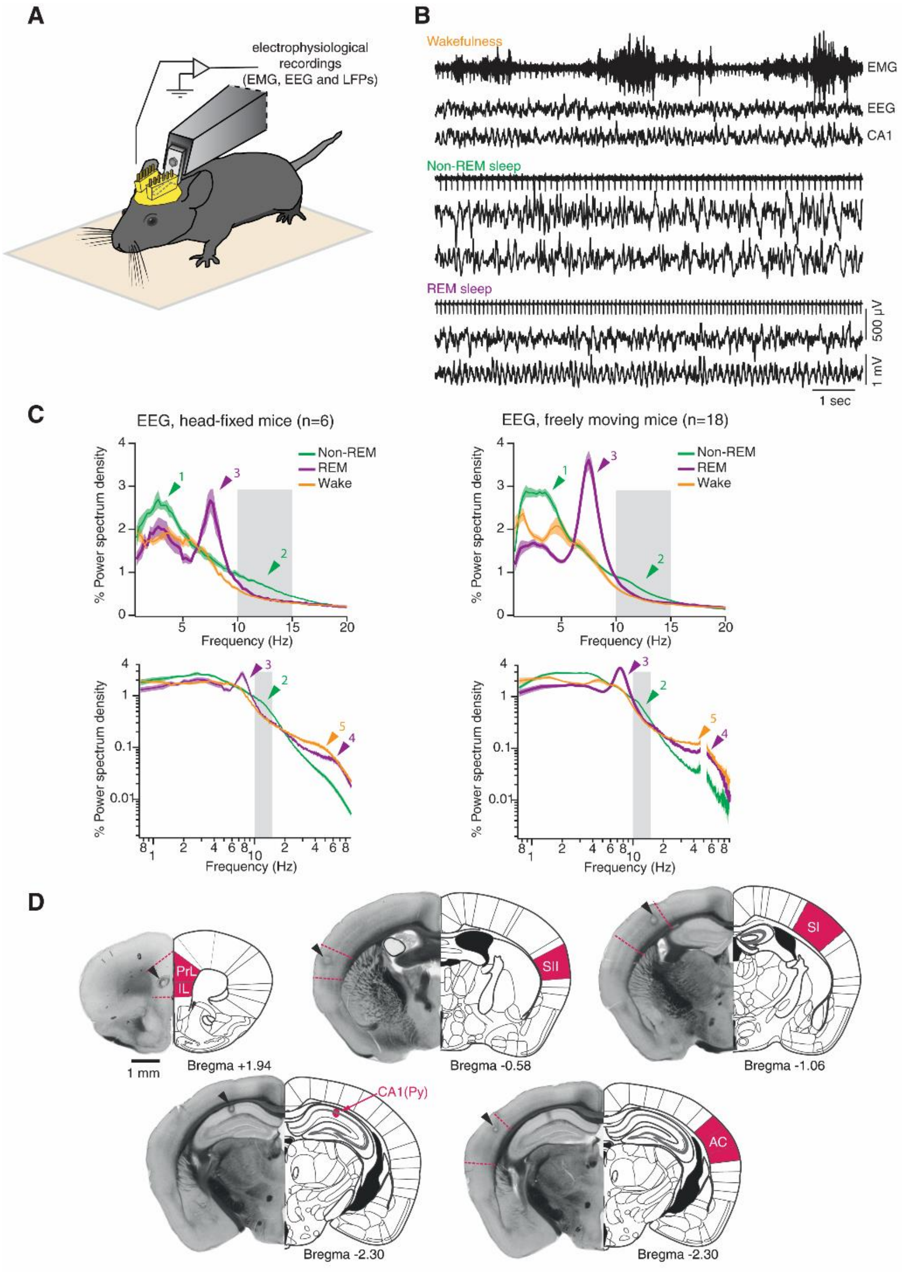

fig. S8. Sleep in head-fixed animals reproduces the three major vigilance states and their spectral characteristics found in freely moving animals. (**A**) Scheme of recording setup. (**B**) Raw EMG (top), EEG (middle) and CA1 LFP traces from wakefulness, non-REM sleep and REM sleep in a head-fixed mouse. Note elevated muscle activity in wakefulness compared to non-REM and REM sleep in the EMG signal. Vertical deflections result from heart beat pick-up (see also Fig. 6). In the EEG, low-frequency high amplitude components are present during non-REM sleep, but not wakefulness or REM sleep. During REM sleep, regular theta waves dominate in the hippocampal LFP trace. (**C**) EEG power spectrum density

derived from recordings in the 6 head-fixed mice (left panels) and the 18 freely moving mice used in Fig. 1 (right panels), with top graphs showing the frequency range up to 20 Hz at an expanded scale. Note the increase in low-frequency and sigma power for non-REM sleep (arrowheads 1, 2) and the theta peak of REM sleep (arrowhead 3) in both recording conditions. Note also the appearance of gamma power for wakefulness and REM sleep (arrowheads 4, 5). Finally, note the similarity in the power values for all vigilance states in both recording conditions. Gray-shaded areas highlight the 10 to 15 Hz sigma power range. (**D**) Location of LFP electrode positions, determined through electro-coagulation at the end of the recordings. Five coronal hemisections with different anterioposterior coordinates (indicated at the bottom of the sections) are shown for a single mouse. Black arrowheads mark electrode locations. The sections are complemented on their right with a schematic drawing of coronal sections according to the Atlas of Paxinos and Franklin (Academic Press, San Diego 2001). Targetted brain areas are labeled in pink. PrL, prelimbic cortex and IL, infralimbic cortex (medial prefrontal cortex), SI, SII, primary and secondary somatosensory cortex, CA1(Py), pyramidal layer of the hippocampal CA1 area, AC, auditory cortex.

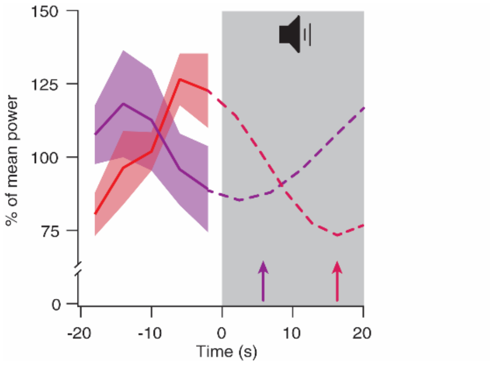

fig. S9. Acoustic stimuli causing early or late wake-ups fall onto late or early portions of the declining sigma power phase, respectively. Time course of sigma power, analyzed separately for whether arousals occurred early (<8 s, purple trace) or late (12 to 16 s, red trace) during noise exposure. The display is the same as in Fig. 4D, showing only the last 20 s of the period before noise exposure (gray-shaded area) are presented. The purple and red arrows indicate the 8-s and 16-s time points, respectively. A total of *n* = 6 animals contributed to early wake-ups, *n* = 9 animals were included for late wake-ups. The putative time course of the 0.02-Hz oscillation is continued into the noise exposure phase according to a 0.02-Hz sine wave (dotted sinusoidal lines). RM ANOVA for factors "time" and "behavioral outcome" yielded $F_{(4,52)} = 2.72$; $P = 0.04$). Data are presented as mean ± SEM.

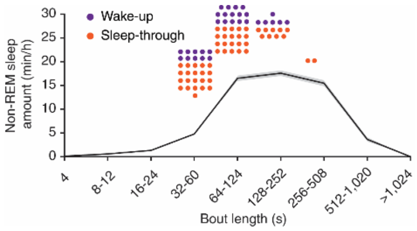

fig. S10. Wake-up and sleep-through trials do not depend on previous sleep duration. Distribution of average non-REM sleep time according to bout duration (subdivided in bins as indicated on x-axis) for *n* = 18 mice, with gray shading indicating SEM. Each color point indicates a single non-REM sleep bout, for which “wake-up” (purple) or “sleep-through” (orange) occurred in response to noise exposure. The color points are positioned within the bin corresponding to their bout duration.

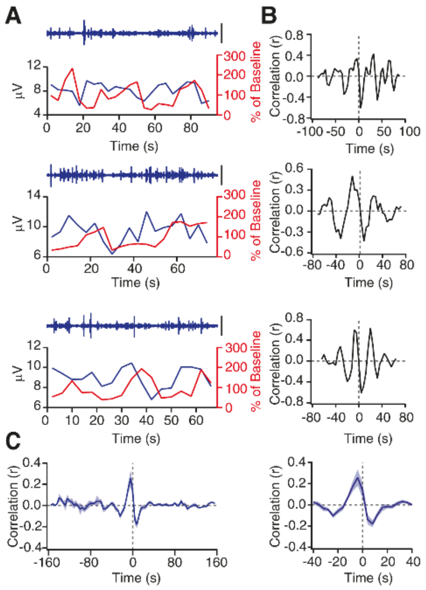

fig. S11. Ripple power increases precede sigma power elevations. (**A**) Three representative traces from CA1 hippocampal LFP recordings filtered between 150 to 250 Hz to extract ripple activity. Together with each filtered raw trace, graphs show the time course of ripple power (dark blue) together with sigma power (red) measured in simultaneous recordings of SI LFP in head-restrained mice. Scalebar: 0.4 mV. (**B**) Corresponding cross-correlograms. (**C**) Left, mean cross-correlograms for *n* = 6 mice. Shading, SEM. Right, expanded portion of the cross-correlogram shows that the positive peak occurs at negative times, indicating that increases in ripple power precede those of sigma power.

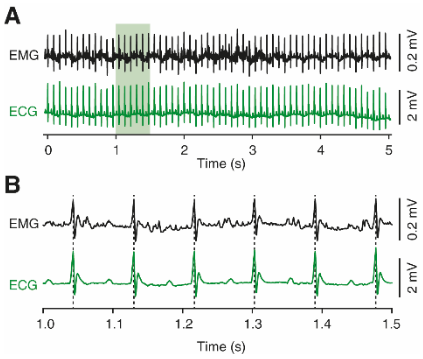

fig. S12. Nuchal EMG recordings faithfully detect the R-waves of the heartbeat in mice. (**A** and **B**) Simultaneous recording of nuchal EMG (black) and ECG (green) in a Urethane-anesthetized mouse (A). Green-shaded portion expanded in B. Note the exact correspondence of R-waves in the two recordings.
